# Improved Interpretability of Brain-Behavior CCA With Domain-Driven Dimension Reduction

**DOI:** 10.3389/fnins.2022.851827

**Published:** 2022-06-23

**Authors:** Zhangdaihong Liu, Kirstie J. Whitaker, Stephen M. Smith, Thomas E. Nichols

**Affiliations:** ^1^Mathematics for Real-World Systems Centre for Doctor Training, University of Warwick, Coventry, United Kingdom; ^2^The Alan Turing Institute, London, United Kingdom; ^3^Wellcome Centre for Integrative Neuroimaging, Functional MRI of the Brain, Nuffield Department of Clinical Neurosciences, University of Oxford, Oxford, United Kingdom; ^4^Nuffield Department of Population Health, Big Data Institute, Li Ka Shing Centre for Health Information and Discovery, University of Oxford, Oxford, United Kingdom

**Keywords:** resting-state functional connectivity, dimension reduction, Canonical Correlation Analysis (CCA), Principal Component Analysis (PCA), interpretability analysis

## Abstract

Canonical Correlation Analysis (CCA) has been widely applied to study correlations between neuroimaging data and behavioral data. Practical use of CCA typically requires dimensionality reduction with, for example, Principal Components Analysis (PCA), however, this can result in CCA components that are difficult to interpret. In this paper, we introduce a Domain-driven Dimension Reduction (DDR) method, reducing the dimensionality of the original datasets and combining human knowledge of the structure of the variables studied. We apply the method to the Human Connectome Project S1200 release and compare standard PCA across all variables with DDR applied to individual classes of variables, finding that DDR-CCA results are more stable and interpretable, allowing the contribution of each class of variable to be better understood. By carefully designing the analysis pipeline and cross-validating the results, we offer more insights into the interpretation of CCA applied to brain-behavior data.

## 1. Introduction

Complex, large-scale health projects, such as the Human Connectome Project (HCP) (Van Essen et al., 2013) and UK Biobank (Sudlow et al., 2015), collect health-related data from cohorts representative of a broad population that makes the neuroimaging data even more valuable.

With such data, a central goal is to understand the interplay between the brain imaging and non-brain imaging variables. Canonical Correlation Analysis (CCA) (Hotelling, 1936; Thompson, 2005) is a widely used tool to study such relationships. CCA and closely related technique Partial Least Squares (PLS), have been applied to many other studies to investigate the links between neuroimaging data and other modalities (Friman et al., 2001; Sui et al., 2010; Krishnan et al., 2011; Grellmann et al., 2015; Smith et al., 2015; Whitaker et al., 2016; Vidaurre et al., 2017; Kumar et al., 2018).

Canonical Correlation Analysis takes in two sets of data and discovers the optimal combination of each set of variables to maximize correlation. To reduce the impact of noise and to avoid a degenerate solution when the number of subjects is less than the number of variables, a dimension reduction is often applied to each dataset and the reduced data are fed into CCA. Smith et al. (2015) and Kumar et al. (2018) applied Principal Component Analysis (PCA) to both behavioral/demographic measures, also known as Subject Measures (SM) and resting-state fMRI (rfMRI) (Brain Measures; BM) to reduce the dimensionality of both to 100. However, those works provided no objective standard on the selection of the dimension to reduce to, and dimension reduction introduces further interpretability issues with the CCA results.

In this paper, we propose an alternative method of dimension reduction: Domain-driven Dimension Reduction (DDR). The main idea is to divide the data into sub-domains by function, then reduce the dimension to each of the sub-domains of SM and BM. Each sub-domain may contain a different number of variables and require different levels of dimension reduction. To this end, we apply a two-way Cross-Validation (CV) method, which estimates the dimensionality automatically by minimizing Predicted Residual Error Sum of Squares (PRESS). We then apply CCA to the DDR-reduced SM and BM to study the correlations between brain and behavior. To further improve the interpretability, orthogonal factor rotation is applied during dimension reduction.

We apply this analysis pipeline to the HCP S1200 release with 1,003 subjects. The performance is assessed by examining canonical correlations, significant canonical variables, and canonical loadings (also known as the structural coefficients). DDR offers us insights into the structure of sub-domains, especially for SM, and more interpretable CCA results. We carefully describe and apply a CV framework to assess the stability of DDR and CCA, by applying a five-fold CV.

For comparison, and to test the stability of the results, we replicated the analysis pipeline used by Smith et al. (2015) (PCA followed by CCA) on the larger S1200 data and then compared its results with DDR CCA.

## 2. Methods

### 2.1. Data

We used *N* = 1, 003 subjects from the HCP S1200 release. For BM, we used the connectivity matrix (partial correlation) generated from resting-state fMRI data. Details of data acquisition can be found in the HCP Reference Manual^1^ and in Smith et al. (2013). For non-imaging data, we considered 234 behavioral and demographic measures and referred them as SM.

#### 2.1.1. BM Pre-Processing

To generate BM, we used the pre-processing on rfMRI as described in Smith et al. (2015), and the pre-processed BM is available for download. We used the same pre-processing pipeline to better compare with their results. In brief, Group-Independent Component Analysis (ICA) was performed to parcellate the brain using a 200-dimensional ICA parcellation. Each subject's rfMRI data was then regressed against this to obtain one time series per ICA region. A functional connectivity matrix for each subject was generated by calculating the Tikhonov-regularized (Tikhonov, 1963) partial correlation for every pair of the time series. This resulted in a 200 × 200 connectivity matrix for every subject, and each of the entries represents a connectivity edge between two ICA regions.

#### 2.1.2. Sign-Flipping to Maximize SM Alignment

To facilitate interpretation of the variable loadings produced by CCA, we flipped the signs of some SM variables to provide a consistent meaning, specifically so that more positive values corresponded to “better” life measures/outcomes. We first selected a benchmark variable “income” and flipped variables that have negative correlations with it; we then examined the definition of each variable, flipping variables that did not already correspond to positive life outcomes. Note that flipping the signs of variables neither change the magnitude of covariance nor the eigenvalues, therefore does not affect the dimension reduction and CCA (proofs are shown in Theorem 2 and 3 in Supplementary Material 2.1). Refer to Supplementary Figure 1 for correlation matrices before and after sign-flipping.

#### 2.1.3. Quality Control and De-Confounding

We removed ill-conditioned SM variables according to three criteria: if they had more than 50% missing values; if the standard deviation was 0; if more than 95% of the total entries were identical values. This left us with 234 SM variables (refer to Supplementary Material 1 for a full list of SM variables after quality control).

Both datasets were normalized by rank-based inverse normal (Blom, 1958) transformation (Beasley et al., 2009) and then de-confounded. Fifteen confounding variables were carefully chosen as those that could potentially affect the relationship between brain and behavior, including age, gender, height, weight, and rfMRI head movement; and squared values for some of these variables such as age and BMI (refer to Supplementary Material 3 for the full list of confounders). De-confounding was applied identically to each set of imaging and non-imaging variables, obtained by the residuals from a linear regression on the confound variables.

#### 2.1.4. Grouping of SM and BM Into Sub-Domains

All 234 SM variables were grouped into 14 sub-domains: alcohol use, alertness, psychiatric history, tobacco use, drug use, emotion, cognition, family history, physical health, motor, personality, sensory, female health and demographics (including SES); this grouping followed the official HCP variable dictionary (https://wiki.humanconnectome.org/display/PublicData/HCP+Data+Dictionary+Public-+Updated+for+the+1200+Subject+Release). BM was grouped based on the 200 different ICA regions mentioned above. Thus, there are 200 BM sub-domains and each contains 200 brain edges. Each brain edge appears twice, one per (two) linked ICA regions, but this redundancy is accounted for in the dimension reduction.

### 2.2. Domain-Driven Dimension Reduction

**Figure 1:**
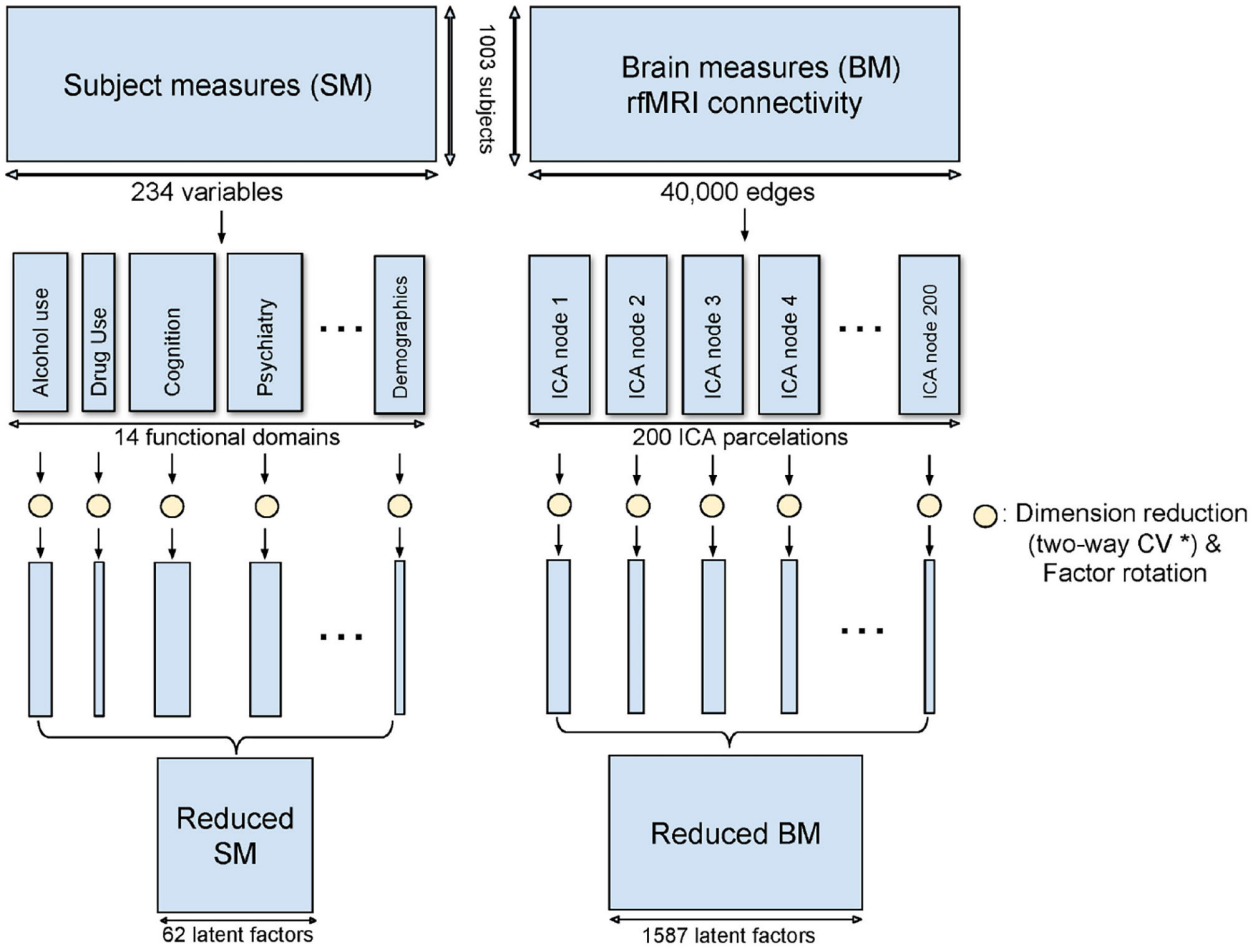
Method overview of DDR. SM and BM are first grouped into sub-domains. PCA is applied to each sub-domain while a two-way CV method (* refer to Figure 2) is used to estimate the dimension. The rotated principal components from all sub-domains are concatenated to form the reduced SM and BM.

**Figure 2:**
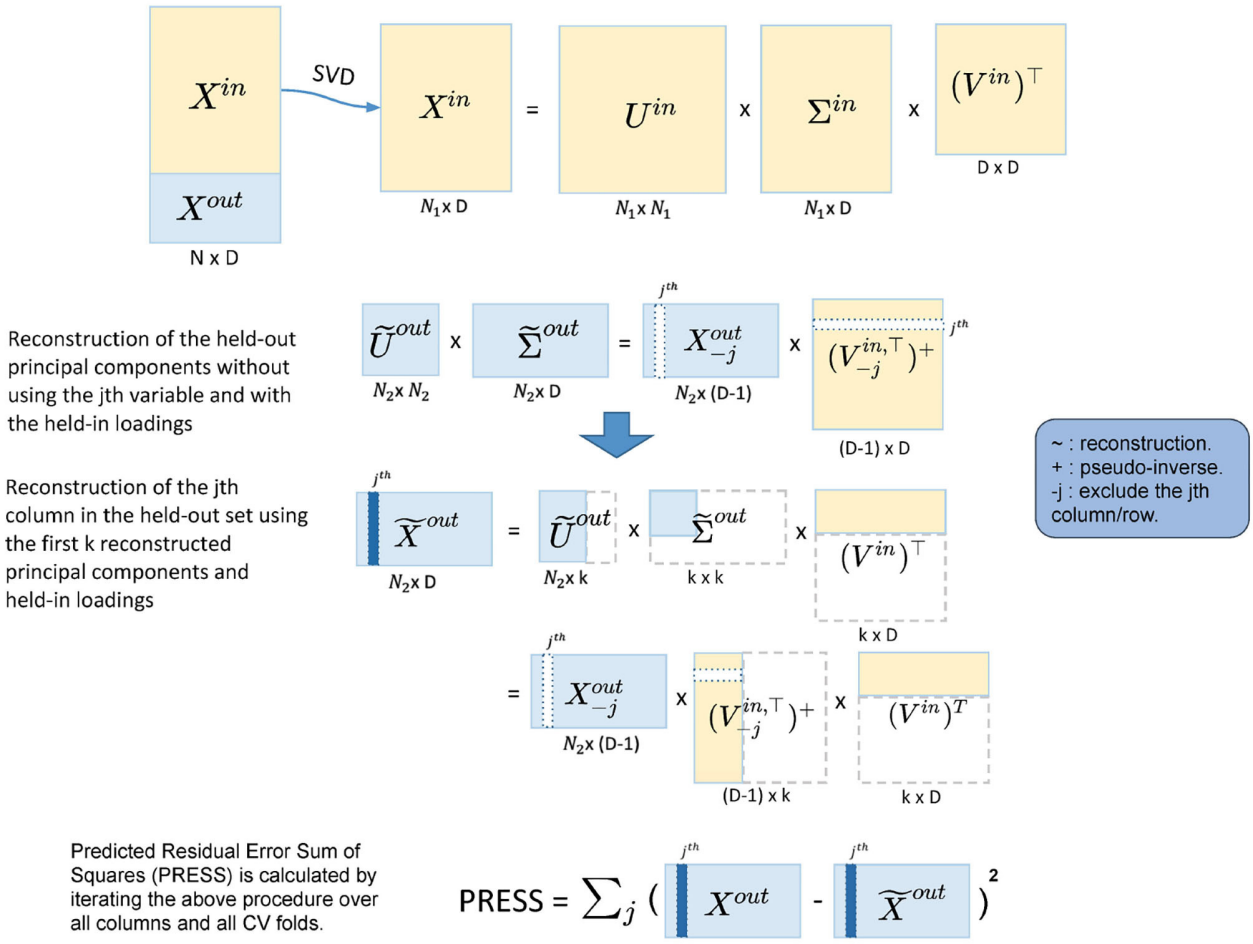
Illustration of the five-fold two-way cross-validation (CV). It minimizes PRESS and estimates the dimensionality in an automated fashion. Yellow blocks represent the training data and light blue blocks represent the test data. A two-way CV includes a subject-way (CV over subject direction) and a variable-way (CV over variable direction). The prediction error is calculated by the reconstruction error using different numbers of principal components.

Domain-driven Dimension Reduction is a refined application of Principal Component Analysis (PCA). Instead of reducing the dimension on the whole data space, SM and BM were grouped as above. PCA was then applied to each sub-domain in turn. The Principal Components (PCs) from the sub-domain analysis were concatenated to form the dimension-reduced data space. The dimensionality of the sub-domains are automatically estimated by minimizing the PRESS using a two-way Cross-Validation (CV) method (Bro et al., 2008). An overview of the method is shown in Figure 1, and the illustration of the two-way CV is shown in Figure 2.

#### 2.2.1. Two-Way CV

The cross-validation for dimension reduction is conducted variable-wise and subject-wise: a five-fold subject-wise CV is performed, and within each of the five-folds, a leave-one-out variable-wise CV is implemented. Dimension estimation is achieved by calculating PRESS for each of the held-out predictions [predicting with different dimensionalities, i.e., 1, 2, 3... PC(s)], selecting the dimension with the lowest PRESS as optimal.

In more detail, for the subject-wise five-fold CV, we split the data into the held-in set (4/5 of the cohort), *X*^*in*^ and the held-out set (1/5 of the cohort), *X*^*out*^. Let *P* be the number of total variables in the sub-domain. Then, we apply Singular Value Decomposition (SVD) to *X*^*in*^,


(1) $$\begin{array}{l} X^{in}=U^{in}\Sigma^{in}{\left(V^{in}\right)}^{T}, \end{array}$$

where *U*^*in*^ is the left-eigenvector matrix of *X*^*in*^, the eigenvectors of the subject covariance ($\frac{1}{P}X^{in}{\left(X^{in}\right)}^{T}$); *V*^*in*^ is the right-eigenvector matrix, the eigenvectors of the variable covariance ($\frac{1}{N}{\left(X^{in}\right)}^{T}X^{in}$); and Σ^*in*^ is the singular value matrix. The PCs of *X*^*in*^ are the singular-value-scaled left-eigenvectors, which we denote $U_{PC}^{in}=U^{in}\Sigma^{in}$, so that Equation (1) becomes


(2) $$\begin{array}{l} X^{in}=U_{PC}^{in}{\left(V^{in}\right)}^{T}. \end{array}$$

Noting that $U_{PC}^{in}=X^{in}V^{in}$ are the observations in the PC space. In order to reduce the dimensionality in the PC space to *k* (*k* < *P*), we can apply the following transformation


(3) $$\begin{array}{l} U_{k,PC}^{in}=X^{in}V_{k}^{in}, \end{array}$$

where $U_{k,PC}^{in}$ and $V_{k}^{in}$ are the first *k* columns in $U_{PC}^{in}$ and *V*^*in*^, respectively.

Then, we can likewise transform the held-out data to reconstruct the first *k* held-out PCs with the k-dimensional held-in principal loadings:


(4) $$\begin{array}{l} {\tilde{U}}_{k,PC}^{out}=X^{out}V_{k}^{in}. \end{array}$$

The lower dimensional reconstruction of the held-out data is thus


(5) $$\begin{array}{l} {\tilde{X}}^{out}={\tilde{U}}_{k,PC}^{out}{\left(V_{k}^{in}\right)}^{T}=X^{out}V_{k}^{in}{\left(V_{k}^{in}\right)}^{T}. \end{array}$$

We can now calculate the prediction error as the difference between *X*^*out*^ and ${\tilde{X}}^{out}$. Iterating this algorithm over all five-folds gives the subject-wise action of our two-way CV method, and we get a PRESS of


(6) $$\begin{array}{l} \overset{N}{\underset{i=1}{\sum }}{\left\|{\text{x}}_{i}^{out}-{\tilde{\text{x}}}_{i}^{out}\right\|}^{2}=\overset{N}{\underset{i=1}{\sum }}{\left\|{\text{x}}_{i}^{out}-{\text{x}}_{i}^{out}V_{k}^{in}{\left(V_{k}^{in}\right)}^{T}\right\|}^{2}, \end{array}$$

where **x**_*i*_ is a row vector and represents the *i*th subject in the held-out set.

However, the PRESS in Equation (6) monotonically decreases as *k* (the number of PC) increases and so is not suitable for dimensionality estimation. This is because the reconstruction of *X*^*out*^ in Equation (6) uses *X*^*out*^ itself. To address this, we modify the reconstruction of ${\tilde{X}}^{out}$ in Equation (5), predicting the *j*th column of *X*^*out*^ using the rest columns in *X*^*out*^:


(7) $$\begin{array}{l} {\tilde{X}}^{out}=X_{-j}^{out}{\left[V_{-j,k}^{in,T}\right]}^{+}{\left(V_{k}^{in}\right)}^{T}, \end{array}$$

where $X_{-j}^{out}$ is *X*^*out*^ with the *j*th column removed; ${\left[V_{-j,k}^{in,T}\right]}^{+}$ is the pseudo-inverse of the transpose of $V_{-j,k}^{in}$, where $V_{-j,k}^{in}$ takes the first *k* columns of *V*^*in*^ and then removes the *j*th row. The pseudo-inverse is required since removing a row of *V*^*in*^ breaks its orthogonality. The *j*th column in ${\tilde{X}}^{out}$ is now reconstructed without using the *j*th column in *X*^*out*^, and we denote this column as ${\tilde{\text{x}}}_{j}^{out}$. If we iterate *j* from 1 to *P*, we reconstruct the whole held-out set in turn. This is the variable-wise action in the two-way CV method. For each of the held-out in a CV fold, the corresponding PRESS can be calculated as


(8) $$\begin{array}{l} \overset{P}{\underset{j=1}{\sum }}\|{\text{x}}_{j}^{out}-{\tilde{\text{x}}}_{j}^{out}\|, \end{array}$$

where ${\text{x}}_{j}^{out}$ is the *j*th column in *X*^*out*^, and ${\tilde{\text{x}}}_{j}^{out}$ is as described above. Finally, the total PRESS for all subjects is calculated by summing PRESS in Equation (8) over all CV folds, completing the subject-wise action of the method.

Finding the dimensionality *k* with the minimum PRESS over dimensions completes the method for a given sub-domain. The reduced datasets for SM and BM are then obtained by the concatenation of the selected PCs from each of the sub-domains.

### 2.3. Evaluating the Stability of DDR

Since DDR is based on CV, different random folds will give different PRESS values. Therefore, we repeated the two-way CV for DDR 50 times and took the mode of the estimated dimension for each sub-domain.

To further test the accuracy/rationality of the dimension DDR estimates, we compared the results from DDR with eigen-spectrum and null eigen-spectrum on each of the sub-domains. The eigen-spectrum provides information on the variance explained (VE) by each of the eigenvectors. The null eigen-spectrum is obtained by shuffling the row values for each column independently in the original matrix, and calculating the eigen-spectrum of the shuffled matrix. It shows the amount of “background noise” that exists in the dataset. When the null eigen-spectrum exceeds the eigen-spectrum, we can interpret this as the background noise taking over the information. If the estimated dimension from DDR falls near where the null eigen-spectrum crosses the eigen-spectrum, we have convergent evidence for the dimensionality estimation.

### 2.4. CCA on Brain Imaging and Behavioral Data

Canonical Correlation Analysis is a multivariate statistical approach to infer the relationship between two sets of variables. It aims to construct latent factors that maximize the correlation between two sets of data, *X* and *Y*, with a common number of rows and possibly different numbers of columns. For column vectors *A* and *B*, CCA finds two sets of linear combinations *P* = *XA* and *Q* = *YB* that are maximally correlated with each other. *P* and *Q* are known as the canonical variables; *A* and *B* are the canonical weights for *X* and *Y*, respectively (Borga, 2001). The correlation between *P* and *Q* is called the canonical correlation, *R*.

To evaluate the importance of variables, we use canonical loadings, also known as structural coefficients (Borga, 2001; Egloff et al., 2010), defined by correlating canonical variables with the observed datasets, in this case, SM (denoted as *X*) and BM (denoted as *Y*):


(9) $$\begin{array}{l} CL_{SM}=corr\left(P,X\right),\\ CL_{BM}=corr\left(Q,Y\right). \end{array}$$

Permutation testing is used to test the significance of the canonical variables.

#### 2.4.1. DDR CCA Pipeline

Since we found that the DDR dimension for BM is still larger than the number of subjects and much larger than the DDR estimation for SM, we applied PCA to DDR-reduced BM to further reduce its dimensionality. We reduced the dimension of BM to 100 to match the method in Smith et al. (2015) and also considered the same dimension as the DDR-reduced SM.

To further improve the interpretation of these PCs, we applied Varimax factor rotation (Kaiser, 1958) to the principal loadings in the sub-domains. The rotated PCs, *RC*_*X*_ and *RC*_*Y*_, are then fed into CCA. Notably, orthogonal rotation is an invariant transformation on CCA inputs. Therefore, it does not affect CCA outputs, only improve the interpretation and robustness of the DDR factors by simplifying the loading structure (Abdi, 2003).

We examined the number of significant pairs of canonical variables using permutation testing and evaluated the variable importance by two different measures: canonical loadings for observed variables, and canonical loadings for DDR factors (CCA inputs). For the first measure, we calculated the same loadings as in Equation (9). The second set of loadings offers insights into the importance of each sub-domain, and are calculated as


(10) $$\begin{array}{l} CL_{SM}=corr\left(P,RC_{X}\right),\\ CL_{BM}=corr\left(Q,RC_{Y}\right). \end{array}$$

We have also calculated VE by each of the significant canonical variables in the original datasets of SM and BM. The R-squared value was computed for each variable and then averaged.

### 2.5. Stability Study of CCA

**Figure 3:**
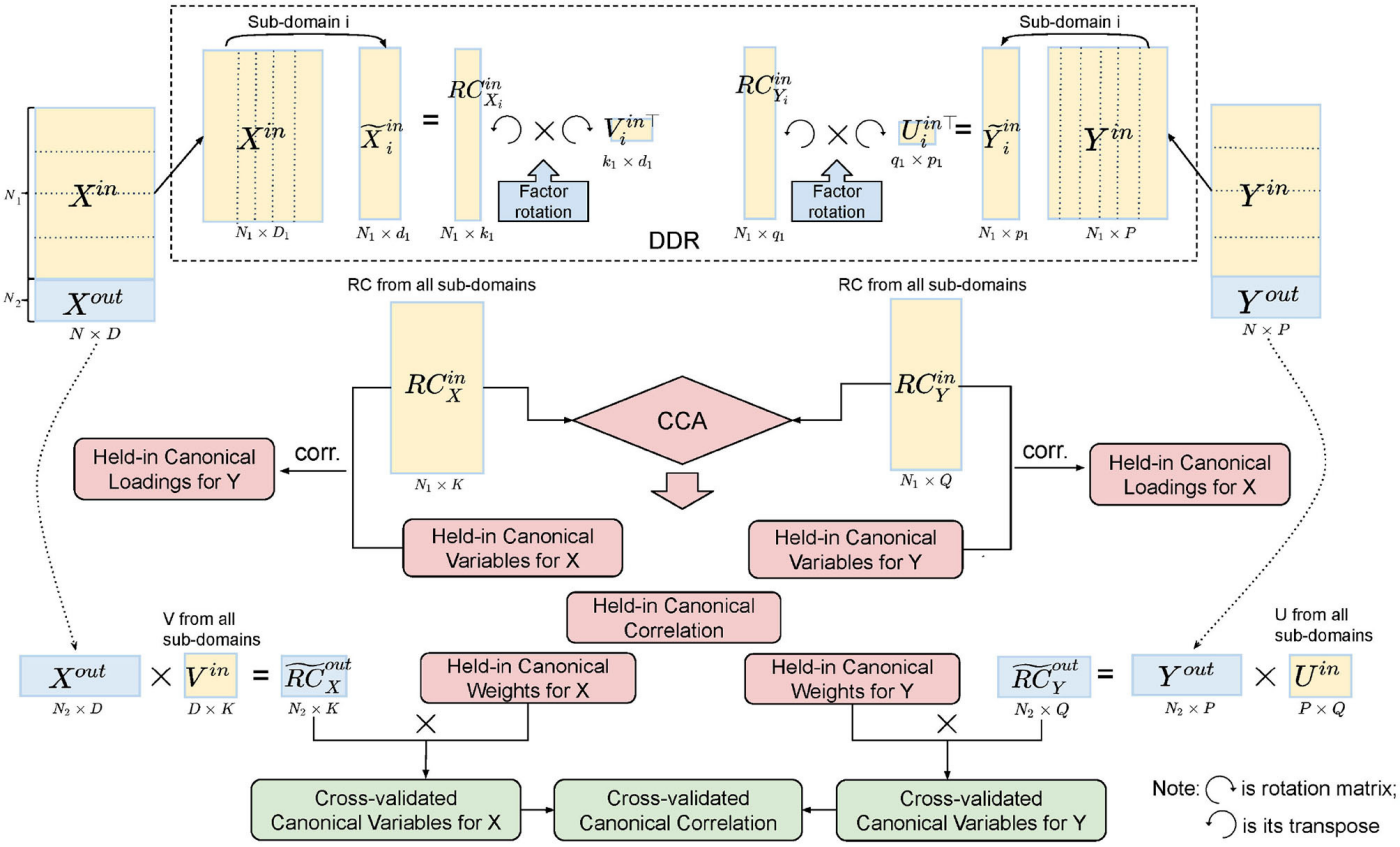
Illustration of five-fold CV in the DDR CCA analysis. We apply DDR (dotted box on the top) to the training set after the split of the data. Within DDR, the principal loadings are rotated to construct the rotated components (RCs), and the RCs from the training set are fed into CCA. Cross-validated canonical variables and correlations are obtained by multiplying the training canonical weights with the RCs from the test set.

In order to test the stability of the CCA results, we applied a five-fold CV (Figure 3). For each fold, we tested our model on the training set, four-fifths of the data (~800 subjects), and validated it on the test set, one-fifth of the data (~200 subjects). The splits do not break the families, i.e., subjects from the same family will go into the same group. The detailed procedure of CV is shown in Algorithm 1.

**Algorithm 1:** Cross-validation procedure for the DDR CCA analysis

1. Step 1: Split the original SM and BM into five roughly equal-sized groups without breaking the families, using four as the training set (*X*^*in*^, *Y*^*in*^) and the other as the test set (*X*^*out*^ and *Y*^*out*^).
2. Step 2: Apply DDR to the training set and use the rotated principal loadings from the training set to construct the rotated principal components for the test set (${\tilde{RC}}_{X}^{out}$ and ${\tilde{RC}}_{Y}^{out}$ in Figure 3).
3. Step 3: Feed both DDR-reduced training set from SM and BM ($RC_{X}^{in}$ and $RC_{Y}^{in}$ in Figure 3) to CCA to obtain canonical variables *P*^*in*^ and *Q*^*in*^, canonical weights *A*^*in*^ and *B*^*in*^ and canonical correlations *R*^*in*^.
4. Step 4: Construct canonical variables for the test set using canonical weights from the training set using Equation (11) (11) $$\begin{array}{l} {\tilde{P}}^{out}=X^{out}A^{in},\\ {\tilde{Q}}^{out}=Y^{out}B^{in}. \end{array}$$
Step 5: Calculate correlations between ${\tilde{P}}^{in}$ and ${\tilde{Q}}^{in}$ as the cross-validated canonical correlations ${\tilde{R}}^{in}$.
5. Step 6: Apply permutation testing to test the number of significant canonical variables for *P*^*in*^ and *Q*^*in*^, and ${\tilde{P}}^{in}$ and ${\tilde{Q}}^{in}$ with 10,000 permutations.
6. Step 7: Compute the assessment measurements such as VE and canonical loadings.
7. Step 8: Go to Step 1 to start the next fold.

### 2.6. Comparison Between PCA and DDR

To assess the performance of DDR in comparison with PCA, we applied the same analysis pipeline using PCA instead of DDR on the same datasets. Smith et al. (2015) applied PCA to reduce the dimensions of SM and BM to both 100. However, our DDR method automatically reduces the dimension of SM to under 100, and of BM to over 100. In order to make the dimensions consistent, we apply PCA to match the dimension of the DDR-reduced SM dataset. For BM, we chose to apply PCA after DDR to reduce the BM dataset to 100.

We compared the VE by the PCs obtained by PCA and DDR, respectively, in the original SM and BM spaces. Feeding PCA-reduced datasets and DDR-reduced datasets into CCA separately to compare their canonical variables, canonical correlations, and canonical loadings, and the VE by canonical variables in the original SM and BM spaces. We also applied the same CV procedure (as shown in Algorithm 1 with the replacement of PCA with DDR) to compare the stability of PCA CCA with DDR CCA.

## 3. Results

### 3.1. PCA-Based CCA

Table 1 shows VE by the significant canonical variables of SM and BM and their canonical correlations at 5 sets of different input dimensions. We notice that the first canonical variable does not necessarily explain the most variance in the observed datasets. Interestingly, Table 1 also shows that if we decrease the dimension(s) of SM and/or BM, the canonical variables would explain more variance in the observed dataset(s). For example, by observing the last three rows in Table 1, the input dimension of BM decreases from 100 to 30. The “VE (%) by BM Canonical Variable” increases for all significant canonical variables. However, the strength of canonical correlation decreases as the dimension decreases.

**Table 1:** Summary table for Principal Component Analysis (PCA) Canonical Correlation Analysis (CCA) with 5 different input dimensions of CCA (first column).

**CCA input dimensions**		**VE (%) by SM canonical variable**				**VE (%) by BM canonical variable**				**Canonical correlation**			
**SM**	**BM**	**CCA1**	**CCA2**	**CCA3**	**CCA4**	**CCA1**	**CCA2**	**CCA3**	**CCA4**	**CCA1**	**CCA2**	**CCA3**	**CCA4**
62	100	3.586	3.817	1.622	0.920	0.205	0.203	0.229	0.199	0.674	0.637	0.604	0.588
62	62	3.296	2.333	1.501	1.207	0.233	0.208	0.266	0.298	0.625	0.543	0.531	0.511
30	100	3.943	4.358	2.216	1.482	0.207	0.191	0.221	0.214	0.649	0.603	0.548	0.514
30	62	3.663	3.534	1.840	1.754	0.234	0.231	0.280	0.278	0.596	0.505	0.458	0.446
30	30	2.869	2.384			0.308	0.370			0.466	0.387		

*The second and third columns show the Variance Explained (VE) by the SM and BM canonical variables in the observed SM and BM datasets for significant canonical pairs, respectively; the fourth column shows the canonical correlation for the canonical pairs; the last column shows the number of significant canonical pairs obtained by permutation testing*.

For the rest of the paper, we focus on the 62 dimensional SM and 100 dimensional BM since 62 is the DDR estimated dimension for SM and 100 was selected in previous studies (Smith et al., 2015). There are 4 significant canonical pairs identified by permutation testing in this setting. The canonical loadings of SM (Supplementary Figure 2) for these 4 canonical variables display 4 behavioral/demographic modes. The first set is mainly loaded on cognition variables; the second set is dominated by tobacco variables; most of the top loadings in the third set are alcohol variables; the fourth set is more of mixture with cognition, emotion, and motor variables.

Note that most of the SM variable loadings have the same sign (Supplementary Figure 2), and this is in contrast to previous CCA results with the HCP data. For example, Smith et al. (2015) found a mode with tobacco use and education measures having opposing signs, while here, after flipping the signs of the observed variables, they are now on the same side of the axis (CCA mode 2 in Supplementary Figure 2). While the canonical variable found by CCA is invariant to sign flips of the variables, the canonical loadings of course reflect any sign flips (refer to Theorem 3 in Supplementary Material 2.1).

### 3.2. DDR Results

We generated a summary report for each of the 14 sub-domains to help us understand the structure of each sub-domain (a full list of reports is attached in Supplementary Material 5).

**Figure 4:**
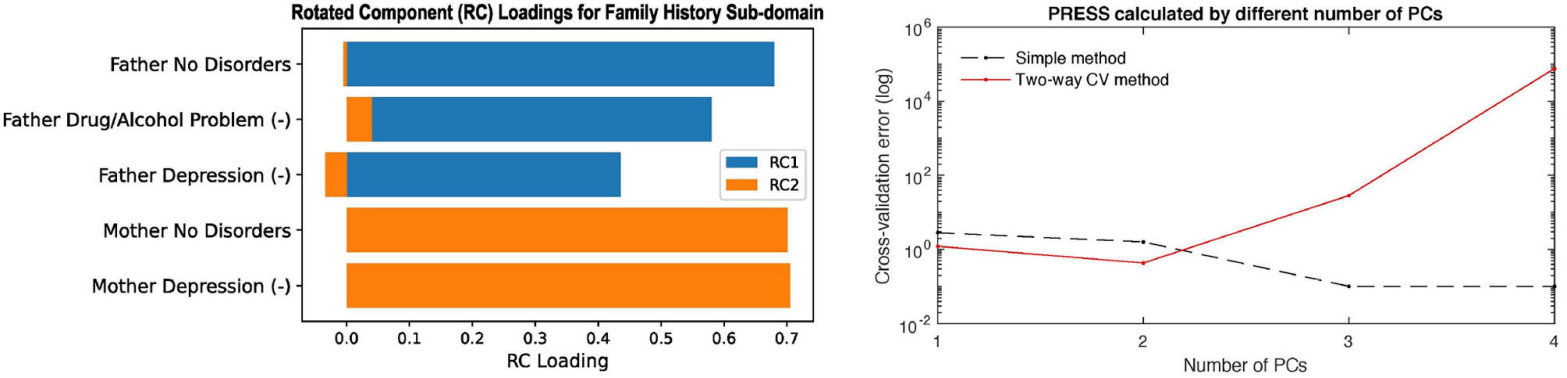
The left panel shows the rotated principal loadings. Variable name with “(-)” indicates it was sign-flipped; the right panel shows the error curves calculated by Equation (6) (dotted line) and Equation (8) (red line), with the minimal error circled at the second component. The naive way of calculating PRESS (dotted line) is monotonically decreasing, while the two-way CV method (red line) offers a minimum point.

Two of the panels in the Family History report are shown in Figure 4 to show as an example. The left panel in Figure 4 shows the rotated principal loadings, i.e., the variable importance in generating the latent factors for the sub-domain. We observed higher interpretability on the rotated loadings: before rotation, the loadings were more evenly distributed across the five variables (Supplementary Figure 3D); after rotation, we can see that “RC1” (blue) is mainly loaded on the father's side of the history and “RC2” (orange) represents the mother's side (left panel in Figure 4). Therefore, we used the rotated loadings to summarize the meaning of the latent factors in the sub-domain, as shown in Table 2. The dimension of the sub-domains is decided by the minima of the red line in the right panel in Figure 4, which is calculated by Equation (8). These DDR estimations also generally corresponded to where the actual and null eigenspectrum cross (Supplementary Figures 3A–16).

**Table 2:** Summary of Subject Measure (SM) sub-domain factors.

**Sub-domain factors**	**Factor summary**	**VE % & DDR estimation**
Demographics	SES	23.85% (1/7)
Physical health 1	Hematocrit	62.53% (3/8)
Physical health 2	Blood pressure	
Physical health 3	BMI (-)	
Female health 1	Regular cycle	100% (5/5)
Female health 2	Days since last cycle	
Female health 3	Cycle length	
Female health 4	Age began menstruation	
Female health 5	Using birth control	
Family history 1	History of mental health disorder - Father (-)	77.05%
Family history 2	History of mental health disorder - Mother (-)	(2/5)
Psychiatry 1	Anxiety (-)	
Psychiatry 2	Attention deficit (-)	
Psychiatry 3	Thought problems (-)	
Psychiatry 4	Aggressive behavior (-)	
Psychiatry 5	Anti-social behavior (-)	86.88%
Psychiatry 6	Withdrawn/avoidant behavior (-)	(10/44)
Psychiatry 7	Somatic (-)	
Psychiatry 8	Intrusive behavior (-)	
Psychiatry 9	Depression (-)	
Psychiatry 10	Panic/phobia (-)	
Sensory 1	Visual acuity (number of errors)	
Sensory 2	Visual and auditory acuity (-)	
Sensory 3	Taste intensity (-)	
Sensory 4	Olfactory ability	90.56%
Sensory 5	Subjective pain experience (-)	(7/12)
Sensory 6	Eyesight	
Sensory 7	Visual acuity and audition	
Drug use	All drug use (-)	46.14% (1/11)
Alcohol use 1	Alcohol abuse and dependence	68.36% (5/28)
Alcohol use 2	Heavy alcohol consumption	
Alcohol use 3	Alcohol consumption (-)	
Alcohol use 4	Hard liquor consumption (-)	
Alcohol use 5	Wine consumption (-)	
Tobacco use	Smokes tobacco (-)	80.54% (1/10)
Alertness	Sleep quality	35.28% (1/9)
Cognition 1	Delay discounting (small amount)	
Cognition 2	Delay discounting (large amount)	
Cognition 3	Delay discounting (short term)	
Cognition 4	Language	
Cognition 5	Fluid intelligence	
Cognition 6	Sustained attention (specificity)	
Cognition 7	Sustained attention (sensitivity)	85.01%
Cognition 8	Executive function - set shifting	(14/44)
Cognition 9	Visuospatial processing	
Cognition 10	Executive function - inhibition (Flanker)	
Cognition 11	Working memory	
Cognition 12	Processing speed	
Cognition 13	Visual episodic memory	
Cognition 14	Verbal episodic memory	
Emotion 1	Social support	
Emotion 2	Negative affect (angry, fearful, sad)	
Emotion 3	Positive affect	
Emotion 4	Aggressive behavior	69.96%
Emotion 5	Emotion recognition (fear and sad)	(8/23)
Emotion 6	Emotion recognition (anger against fear)	
Emotion 7	Emotion recognition (neutral against sad)	
Emotion 8	Emotion recognition (fast response time)	
Motor 1	Endurance	86.84%
Motor 2	Strength	(3/7)
Motor 3	Dexterity	
Personality	N against ACE	39.70% (1/5)

*The factors are orthogonally rotated principal components and ordered by R-squared values in the original sub-domain. The second column shows the factor names summarized from sub-domain reports Supplementary Figures 3E–16E. The third column shows the VE by the dimension reduced sub-domain in the original sub-domain. The numbers in brackets are the two-way CV estimated dimension vs. the total number of variables in the sub-domain. Personality contains the Big Five personality traits: neuroticism (N), agreeableness (A), extraversion (E), conscientiousness (C), openness to experience (O)*.

By investigating the sub-domain structures, we observed strong stability of the DDR factors, the amount of VE by the factors in the test set having matched level with the training set (Supplementary Figures 3B–16). Moreover, we understood better the composition of the latent factors in each sub-domain.

In total, DDR selected 62 SM factors from the 14 sub-domains. Different from choosing PCs by a simple cut-off point from the VE point of view, we can see factors in different domains explain the different amount of variances (third column, Table 2). For example, the first PC (out of 10) in Tobacco Use explains 80.54% variance in the whole sub-domain, and the first PC (out of 11) in Drug Use explains less than 50%. However, with only 1 PC in these two sub-domains, they achieved the lowest prediction errors in the test set. For BM, DDR reduced the 200 ICA regions/sub-domains from a total dimension of 40,000 to 1,587.

### 3.3. DDR-Based CCA

Similar to the PCA-based CCA analysis, we applied DDR-based CCA analysis to different input dimensions of CCA (Table 3). The same rule in PCA-based CCA was found here: lower CCA input dimension leads to canonical variables explaining more variance, whereas the canonical correlations get weaker. Comparing each setting with results in PCA-based CCA (Table 1), we found that the number of significant canonical variables are always one lower than the PCA case.

**Table 3:** Summary table for DDR CCA.

**CCA input dimensions**		**VE % by SM canonical variable**			**VE % by BM canonical variable**			**Canonical correlation**		
**SM**	**BM**	**CCA1**	**CCA2**	**CCA3**	**CCA1**	**CCA2**	**CCA3**	**CCA1**	**CCA2**	**CCA3**
62	100	2.629	1.727	1.698	0.202	0.183	0.211	0.632	0.582	0.574
62	62	2.318	1.386	2.361	0.203	0.302	0.271	0.555	0.519	0.495
30	100	3.323	3.928	1.737	0.194	0.181	0.267	0.586	0.541	0.503
30	62	2.776	2.867	1.290	0.306	0.260	0.244	0.475	0.445	0.440
30	30	2.716			0.376			0.419		

*The first column shows the dimensions of SM and BM as CCA inputs; the second and third columns represent the VE by the SM and BM canonical variables in the observed SM and BM set for significant canonical pairs, respectively; the last column shows the canonical correlation for the canonical pairs*.

#### 3.3.1. Canonical Loadings for SM

**Figure 5:**
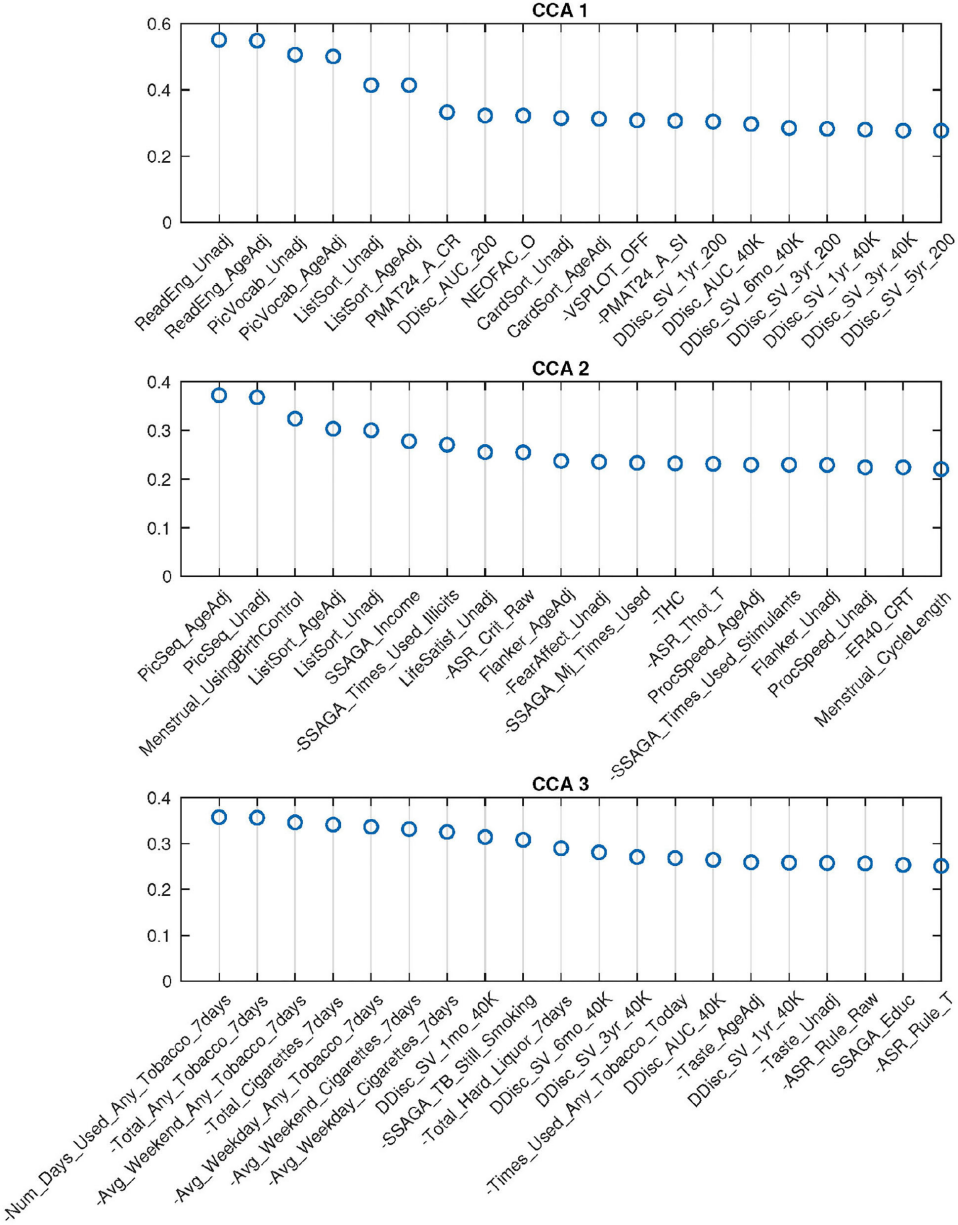
Top 20 SM canonical loadings for 3 significant canonical variables. Variable name with “-” sign shows that it was flipped in the original dataset. Canonical loadings of CCA 1 are very similar to the first set of PCA CCA, heavily cognition dominated; the second set is mixed with cognition, drug use, etc.; The third set is combination of tobacco use and cognition variables.

For 62-dimensional DDR SM and 100-dimensional DDR+PCA BM, permutation testing identified three significant canonical pairs. The top 20 canonical loadings (in absolute value) for each of them are shown in Figure 5. Noticeably, all top 20 loadings for these three canonical variables are positive after sign-flipping of the observed variables.

**Figure 6:**
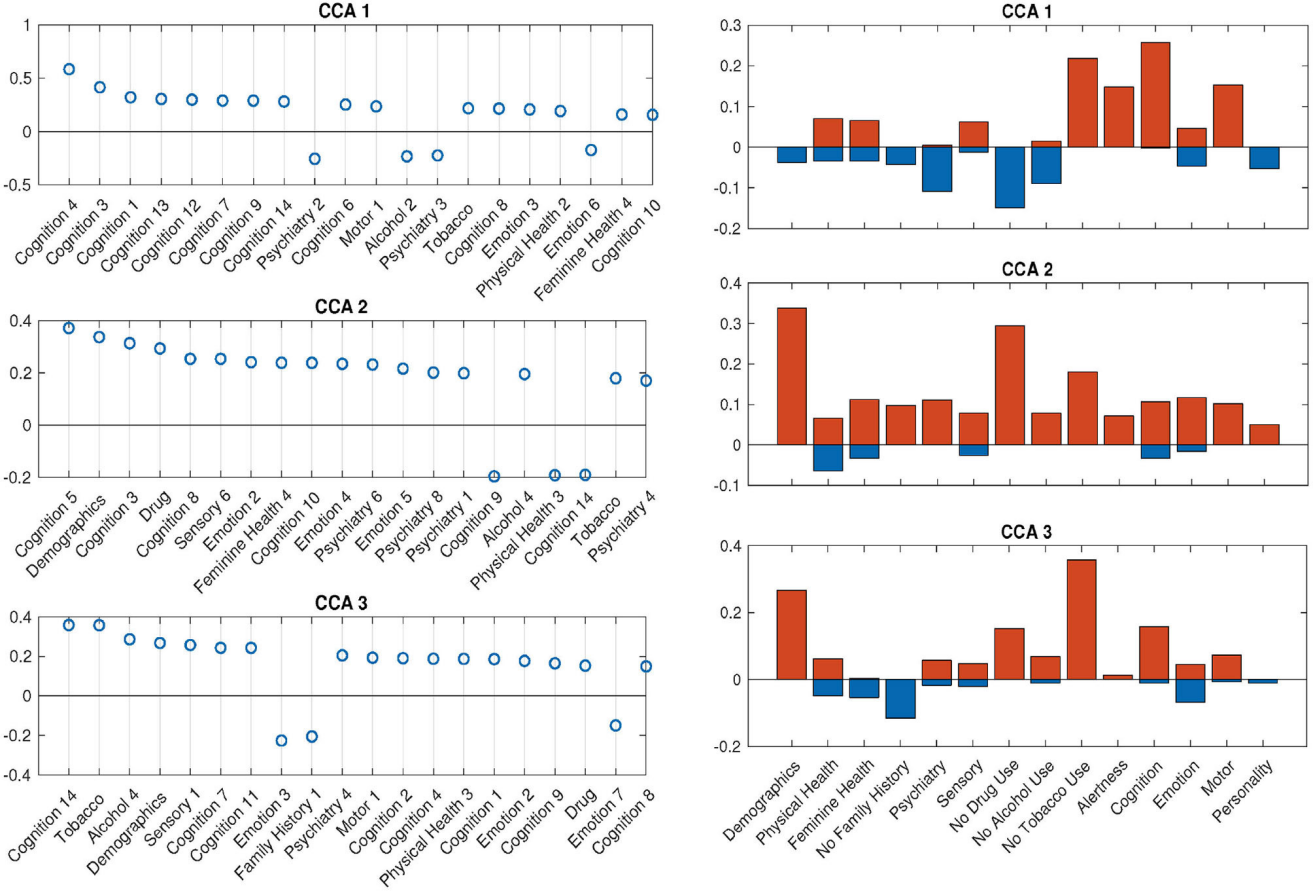
SM canonical loadings on the CCA input for 3 significant canonical variables. The **left** set of figures shows top 20 loadings for 3 significant canonical variables, respectively. One can combine with Table 2 to better understand the factors on the x-axis. The **right** set of figures show the mean of all positive loadings (red bars) and the mean of all negative loadings (blue bars) within each sub-domain for the 3 significant canonical variables.

With the help of DDR, we are able to explore the contributions of CCA inputs directly, by calculating the canonical loadings of them using Equation (10). The canonical loadings of the inputs cannot be directly interpreted in PCA-based CCA. However, with DDR, we are able to interpret not only the latent factors but also the canonical loadings on those factors (Figure 6). Using the summarized latent factors in Table 2, we are able to conclude, for example, in the first set of canonical loadings (the first subplot in Figure 6), the Language factor (Cognition 4) has the largest loading. The second and third largest loadings are Cognition 3 and 1, and they are delay discounting factors.

The right set of figures in Figure 6 offers us insight into the overall contribution of each sub-domain, by the sign of their contribution: the total length of each blue-red bar pair is the average R-squared values, with the contribution from positively-weighted variables plotted above the x-axis, negatively-weighted plotted below. We notice here the top loadings and overall loadings are not mono-signed anymore even with the sign-flipping in effect. Interestingly, the pattern presented in the first overall canonical loadings (top right, Figure 6) is driven by people with good cognition and motor ability, who do not smoke, but take drugs, have some kind of mental disorder and drink. The second and third sets are displaying good wellbeing patterns. In particular, the second set of loadings is dominated by high SES and no drug use; the third set shows the alignment between no tobacco use and high SES. All of these patterns cannot be observed by using PCA after sign-flipping.

#### 3.3.2. Canonical Loadings for BM

**Figure 7:**
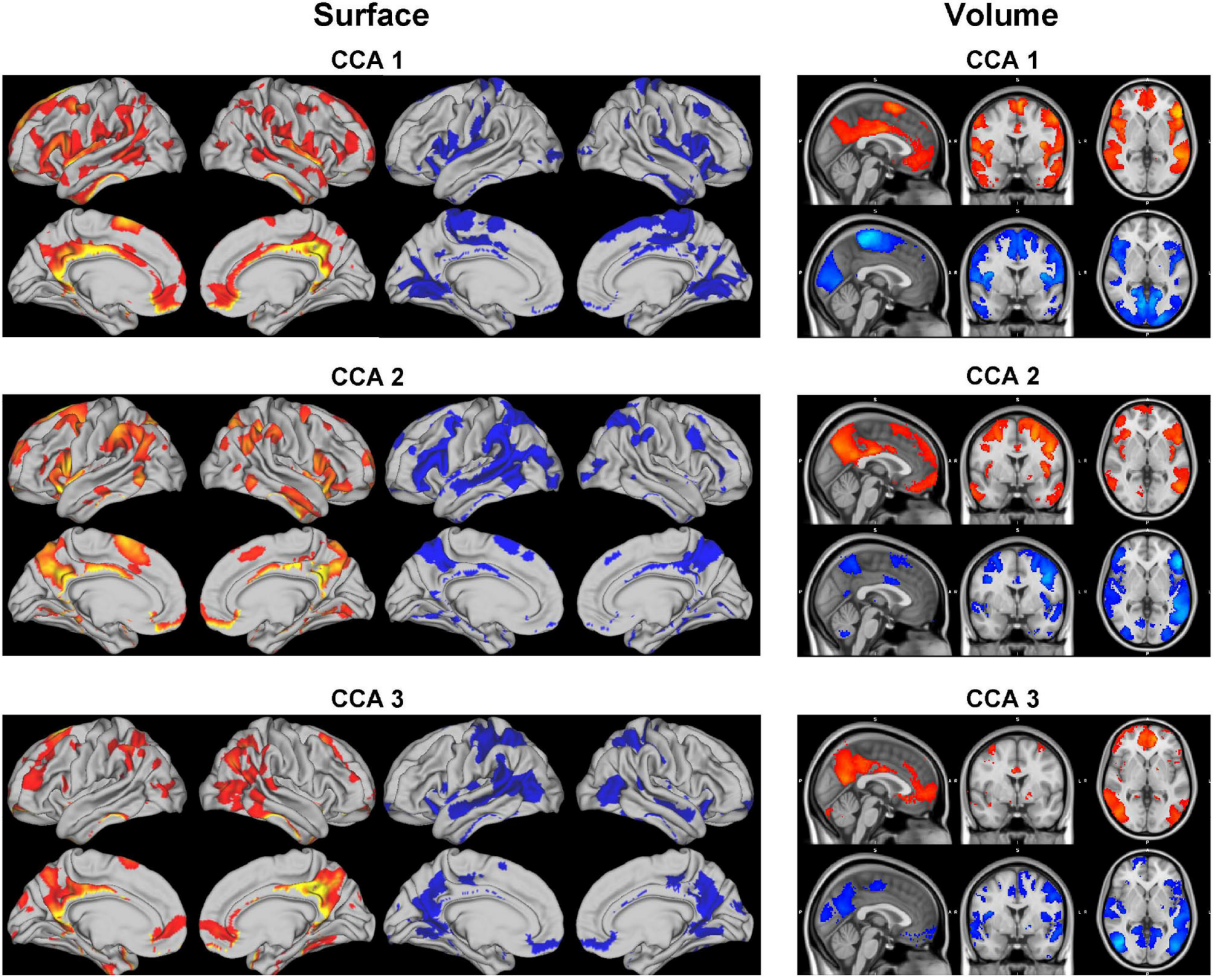
Positive and negative CCA strengths on the brain surface **(left)** and volume **(right)** for the 3 significant canonical variables. The visualization is cut by 80%. Positive (red maps) and negative (blue maps) CCA strengths are generated by mapping the canonical loading with the sign of population mean correlation between each pair of ICA regions, then averaging the top 20 positive and negative modulated loadings, respectively.

Each set of canonical loadings for BM is a 200×200 symmetric matrix. Each entry represents a CCA connection (edge) between two ICA regions. We first map this loading matrix with the signs of the group mean correlations between the ICA regions, i.e., if two ICA regions were negatively correlated at the resting-state, it would decrease the positive CCA strength but enhance the negative CCA strength. Due to the difficulty of interpreting each of these 19,900 (200 * 199/2) edges, we came up with the following summary statistics. Averaging the top 20 (10%) positive and negative modulated canonical loadings for each ICA region (in each column/row) as the positive and negative CCA strength, respectively. They are shown in Figure 7.

The CCA strengths for CCA 1^2^ illustrate a weak contrast between language, sentences, semantic areas (positive strength) against premotor, motor, and primary areas (negative strength); positive and negative strengths for CCA 2^3^ are much less distinguishable, both overlapping with parietal and intraparietal which are arguably linked to working memory and default mode network. The positive CCA strength maps for CCA 3^4^ overlaps considerably with CCA 2. The positive map shops weak connection with the default mode network whereas the negative map activates in occipital and pre-motor areas.

Combining results from both SM and BM sides, CCA mode 1 reveals an interesting pattern: language and comprehension-related brain areas associate positively with no tobacco use, no psychiatric illnesses, better alertness and cognitive ability, and negatively with drug use. Whereas drug use is positively correlated with the motor areas in the brain.

### 3.4. Stability of DDR-Based CCA

We applied five-fold CV to DDR CCA with 62-dimensional DDR SM and 100-dimensional DDR+PCA BM. We also ran permutation testing on the training and cross-validated sets for 10,000 simulations to get significant canonical pairs. Permutation testing resulted in mostly 2 significant canonical pairs on the training sets, and ranged from 0 to 4 on the CV sets, with 0 or 1 being the common numbers.

In general, for canonical correlation, as the sample size gets larger, the correlation gets weaker. The first canonical correlation is 0.632 for 1,003 subjects, and 0.662 on average for four-fifths of those subjects (Table 4). This also applies to the number of significant CCA pairs permutation test detects. With the HCP 500 release, only 1 significant pair was detected (Smith et al., 2015); we replicated the study with the HCP 900 release and found 2 pairs; In this study, 3 pairs were discovered using the whole cohort. However in CV, the training set consists four-fifth of the subjects (the same amount as in the 900 release), and 2 is the most common significant number [with the permuted mean canonical correlation being around 0.6, and standard deviation being around 0.01 (Table 4)], which is consistent with the previous finding. Hence, we are going to examine further the results on the 2 significant pairs of training sets in CV.

**Table 4:** Five-fold CV on 62 dimensional SM and 100 dimensional BM in DDR CCA analysis.

	**CCA 1**	**CCA 2**	**CCA 3**
Mean VE (%) in held-in SM set (std)	2.34 (0.50)	2.13 (0.85)	1.95 (0.84)
Mean VE (%) in held-in BM set (std)	0.23 (0.03)	0.22 (0.03)	0.22 (0.02)
Mean VE (%) in CV SM set (std)	2.80 (0.73)	2.60 (0.85)	2.37 (0.57)
Mean VE (%) in CV BM set (std)	0.63 (0.05)	0.65 (0.09)	0.65 (0.06)
Mean canonical correlation for held-in set (std)	0.662 (0.011)	0.639 (0.016)	0.611 (0.008)
Mean canonical correlation for CV set (std)	0.228 (0.037)	0.108 (0.057)	0.174 (0.039)

*Mean VE and canonical correlations are shown for the first 3 pairs of canonical variables with standard deviation (std) in brackets*.

We have selected the top 20 SM canonical loadings in every fold and took the ones that occurred at least two times out of the five-folds CV for the 2 significant canonical variables. Stability of the first and second sets of SM canonical loadings on the observed SM data are shown in Supplementary Figure 17. Language variables turn out to be the most stable and heavily weighted in the first set, appearing on the top in every fold, and most of the variables in the first set in CV appeared in the first canonical loadings for the whole cohort. The second set of canonical loadings turns out to be less stable than the first one with the most occurrence being 3 out of 5. The second set of canonical loadings in Supplementary Figure 17 looks like a combination of the second and third sets in the whole-cohort analysis.

**Figure 8:**
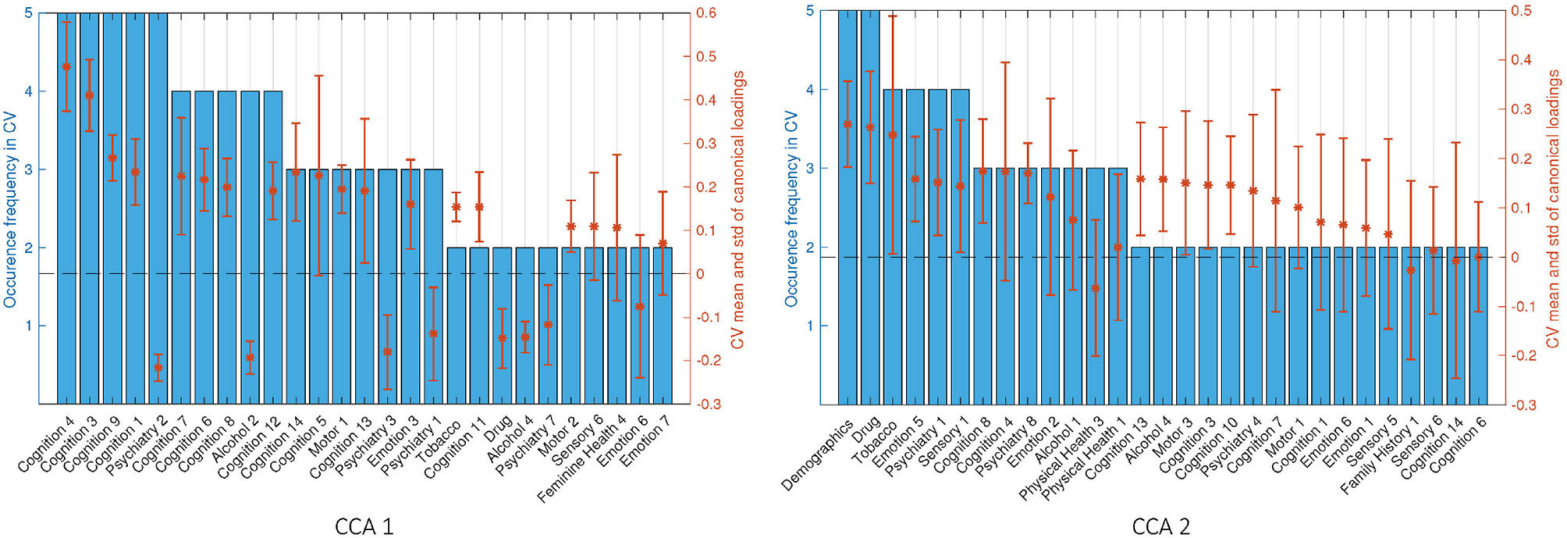
Stability of SM canonical loadings on CCA input. The bar plot shows the occurrence frequency in CV out of the five-folds. Variables are chosen by selecting the top 20 mostly weighted ones in each fold. The ones that appeared at least two times are shown above. The right axis shows the mean and the SD over all occurred loadings. **Left** and **right** plots are the canonical loadings for the first and second canonical variables, respectively.

We focus on the stability of SM canonical loadings on the CCA input (Figure 8). The top two most stable latent factors are the Language factor (Cognition 4) and Delay Discounting (Cognition 3), and they are the top two in the one-off analysis (Figure 6). Similar to the canonical loadings on the observed data, the second loadings here are combined from the second and third loadings in the whole-cohort analysis. Comparing the stability of the canonical loadings on CCA input (Figure 6) with the canonical loadings on observed variables (Supplementary Figure 17), there is an improvement from the occurrence frequency point of view and in the variance of the canonical loadings across different folds. Particularly for the second set of canonical loadings, loadings on the observed variables have the highest occurrence of 3, whereas 5 on the CCA input. The only factor DDR chose in Demographics and Drug Use appeared in every single fold with high and stable canonical loadings, which we cannot observe from the loadings on the observed variables. Moreover, similar to the one-off analysis on the whole cohort, the stability of canonical loadings on the CCA input presents the contrast of the relationship again. Psychiatry, Drug, and Alcohol factors have opposite contributions to Cognition and Motor ones in the first set of canonical loadings (refer to Supplementary Figure 18 for the stability on BM canonical loadings).

## 4. Discussion

In this paper, we carefully replicated the study by Smith et al. (2015) with a modified analysis pipeline for the HCP S1200 release. Comparing with the arbitrary choice of 100-dimension PCA in that work, we proposed a more automated way of estimating the dimensionality of the data, particularly for the function-specific sub-domains of the SM and independent regions of BM. It is often quite challenging to interpret the results of Canonical Correlation Analysis (CCA) applied to behavioral and brain imaging data, e.g., the canonical variables, canonical loadings, canonical correlations, etc. The biggest motivation for proposing DDR is to improve the interpretability of these results.

### 4.1. Sign-Flipping and De-Confounding

Sign-flipping all variables to align with positive life outcomes produced modes that were consistent with Smith et al. (2015), however, when plotted, the results appear different due to sign-flipping. In particular, the canonical loading of variables, like the Picture Vocabulary Test, Oral Reading Recognition Test, Fluid Intelligence (correct response), now shares the same sign as tobacco and alcohol measures (Supplementary Figure 2 and Figure 5).

Aside from sign-flipping, differences from previous HCP results may arise, in part, from the different variable sets used, as this work considered a wider range of variables. Other differences included a slightly different set of confounders, with this analysis using racial factors, release versions, age, and gender as additional confounders.

### 4.2. Comparing DDR With PCA

By grouping the variables of SM into sub-domains based on their functions, we are able to interpret the canonical loadings of the inputs of CCA as shown in Figure 6. This would not be straightforward when using principal components of the whole data space as inputs. By doing so, we have also saved the effort of manually selecting relevant variables that feed into the analysis. We believe that the dimension estimating algorithm we applied minimizes the noise in each of the sub-domains, therefore, achieves the same goal of picking important information manually from each functional domain. Although, the DDR-reduced space would explain less variance than the same dimensional PCA-reduced space as PCA is designed to maximize variances; DDR focuses more on the structure within variables that share the same functionality, making sure each functional domain has a representative number of components feeding into CCA.

Both PCA and DDR have their own advantages and disadvantages. Using data that is reduced by PCA as inputs, the canonical correlations are higher than using DDR (Tables 1, 3). Permutation testing gives more significant canonical variables for PCA and those explain slightly higher variance in the original datasets. This is due to the fact that PCA-reduced sub-space is still orthogonal, whereas DDR-reduced sub-space is not. This allows PCA to capture more variance in the observed dataset than DDR (with the same dimensionality). However, DDR saves the effort of selecting relevant variables manually and it automatically estimates the dimensionality. One of the largest drawbacks of PCA is that the results are not as interpretable as DDR. With DDR, we could track the contribution of each sub-domain and directly interpret the canonical loadings of CCA inputs which cannot be easily interpreted in the PCA case. Moreover, these loadings are not subject to the signs of the observed variables. We applied the same stability analysis to canonical loadings on the DDR factors (CCA inputs) and found higher stability than the loadings on the observed variables (Supplementary Figure 17 and Figure 8).

### 4.3. What We Learn From CCA

In general, we have found that with a larger sample size, CCA tends to find weaker canonical correlations (Tables 1, 3). This is consistent with previous work that showed canonical correlation tends to have a higher bias with a smaller sample size (Lee, 2007). When we try to interpret canonical correlation using small samples, we should be extremely cautious and depend on out of sample validation to obtain unbiased estimates of canonical correlation.

Additionally, canonical correlations get weaker if we use lower dimensional data as inputs. This is explained by higher dimensional data having greater flexibility to maximize the correlation. We observe that canonical variables constructed by lower dimensional data actually have increased average VE in the observed datasets (Tables 1, 3). Further analysis shows that the amount of VE in the original dataset has a non-linear behavior against the CCA input dimension, and it peaks at around dimension 30 in this study.

Further, we found that the mean, median, and 90th percentile of the distribution of canonical loadings also reduced with increased CCA input dimension. Hence, we postulate that higher dimensional inputs may overfit and produce canonical variables that are less related to the original variables.

Since CCA maximizes the correlation between two sets of data rather than the variance canonical variables explain in their original datasets, it is important to be aware that VE can be an informative measure, however, cannot become the sole measure used to assess CCA performance. Other measures should be considered such as canonical loadings and canonical correlations.

### 4.4. Interpretation of CCA Loadings

Variable importance is always a major challenge in interpreting CCA results. The canonical weights are the most direct measures of the importance of CCA inputs. However, they are sensitive to the inputs: small perturbation in inputs can lead to significant change in canonical weights, thus not ideal for variable importance evaluation (Bro et al., 2008; Gittins, 2012). Different studies (Thorndike and Weiss, 1973; Bro et al., 2008) have suggested using structural coefficients which are also known as canonical loadings to measure the variable importance. In this study, we have shown that it is a stable measurement with the canonical loadings on DDR factors being more stable than on observed variables (Figure 8 and Supplementary Figure 17).

Notably, principal components and canonical variables are sign-invariant (proofs shown in Theorems 2 and 3 in Supplementary Material 2.1. Simply flipping the column sign of the inputs of PCA/CCA should not change the principal components/canonical variables. However, this would change the sign of the corresponding canonical loadings since they are just correlations between canonical variables and the CCA inputs. One good example is that after we flipped the signs of the observed variables, the top canonical loadings all have the same signs in Figure 5. The sign contrast, for example, between *Life satisfaction* and *Positive test for THC (cannabis)* that was exhibited in Smith et al. (2015) disappeared (These two variables are “LifeSatisf_Unadj” and “THC”, respectively, in Figure 5 and variable “THC” was sign-flipped). Therefore, one should not interpret the absolute sign of canonical loadings as the positive/negative contribution of the variable. We should interpret the loadings from the variable importance point of view, and take into account the picture on the other side, in our case, linking SM and BM canonical loadings.

Whereas DDR has shown superior ability in improving the understating of SM in brain-behavior CCA, due to the limit of the sample size, we had to accommodate the BM interpretation using an extra PCA step. This is the main limitation to this analysis. In future work, this extra PCA step can be saved by using a larger dataset such as the UK Biobank.

Interpretation of latent factor models like CCA still remains challenging. Researchers often need to trade between model performance and interpretability. However, in the areas of medical/public health research, being able to interpret the results of any statistical/machine learning model is of vital importance. The DDR method proposed here tries to combine prior knowledge on the data collected with mathematical models, to improve the understanding of the intermediate and final results of a CCA pipeline, at the same time, reducing the arbitrary choices researchers have to make and increasing analysis automation. In order to understand totally the mechanism between brain and behavior, and fully interpret the results of all different kinds of latent factor models, much additional research across disciplines is still required.

## Data Availability Statement

Publicly available datasets were analyzed in this study. This data can be found at: https://db.humanconnectome.org/. The code used for the analysis in this paper can be found at: https://github.com/lzdh/DDRCCA_HCP1200release. Further inquiries can be directed to the corresponding author.

## Ethics Statement

Ethical review and approval was not required for the study on human participants in accordance with the local legislation and institutional requirements. The patients/participants provided their written informed consent to participate in this study.

## Author Contributions

ZL, TN, SS, and KW contributed to conception and design of the study. ZL performed the statistical analysis and wrote the first draft of the manuscript. All authors contributed to manuscript edits, revision, read, and approved the submitted version.

## Funding

ZL was funded by the Chinese Scholarship Council, EPSRC, and MRC (Grant Number EP/N510129/1), TN and SS were funded by the Wellcome Trust (Grant Numbers 100309/Z/12/Z and 110027/Z/15/Z), and KW was funded by the EPSRC (Grant Number EP/L015374/1).

## Conflict of Interest

The authors declare that the research was conducted in the absence of any commercial or financial relationships that could be construed as a potential conflict of interest.

## Publisher's Note

All claims expressed in this article are solely those of the authors and do not necessarily represent those of their affiliated organizations, or those of the publisher, the editors and the reviewers. Any product that may be evaluated in this article, or claim that may be made by its manufacturer, is not guaranteed or endorsed by the publisher.

## Footnotes

^1^ https://www.humanconnectome.org/storage/app/media/documentation/s1200/HCP_S1200_Release_Reference_Manual.pdf

^2^Positive map: http://neurosynth.org/decode/?neurovault=108956; negative map: http://neurosynth.org/decode/?neurovault=108957.

^3^Positive map: http://neurosynth.org/decode/?neurovault=108976; negative map: http://neurosynth.org/decode/?neurovault=108977.

^4^Positive map: http://neurosynth.org/decode/?neurovault=108978; negative map: http://neurosynth.org/decode/?neurovault=108979.
