# Supplementary material for: Improved Interpretability of Brain-Behavior CCA With Domain-Driven Dimension Reduction
Source: Front Neurosci. 2022 Jun 23;16:851827. doi: 10.3389/fnins.2022.851827 (PMC9262103; doi:10.3389/fnins.2022.851827)
Supplement: Supplementary file 1 [file Data_Sheet_1.PDF]

Supplementary Material

1 FULL LIST OF SM VARIABLES

The following variables are the 234 subject measures went into the analysis. They are listed using the formal database naming (see `https://wiki.humanconnectome.org/display/PublicData/HCP+Data+Dictionary+Public-+Updated+for+the+1200+Subject+Release` for their detailed descriptions); names with a '-' sign in front indicates the variable is sign-flipped:

Subject, Release, Acquisition, Gender, Age_in_Yrs, Race_white, Race_black, Race_other, Ethnicity, Height, Weight, BMI, Head_motion, fMRI_3T_ReconVrs, FS_IntraCranial_Vol, FS_BrainSeg_Vol, Handedness, SSAGA_Employ, SSAGA_Income, SSAGA_Educ, SSAGA_InSchool, SSAGA_Rlshp, SSAGA_MOBorn, -SSAGA_BMICat, -SSAGA_BMICatHeaviest, Hematocrit_1, Hematocrit_2, BPSystolic, BPDiastolic, ThyroidHormone, HbA1C, Menstrual_RegCycles, Menstrual_AgeBegan, Menstrual_CycleLength, Menstrual_DaysSinceLast, Menstrual_UsingBirthControl, -FamHist_Moth_Dep, -FamHist_Fath_Dep, -FamHist_Fath_DrgAlc, FamHist_Moth_None, FamHist_Fath_None, -ASR_Anxd_Raw, -ASR_Anxd_Pct, -ASR_Witd_Raw, -ASR_Witd_T, -ASR_Soma_Raw, -ASR_Soma_T, -ASR_Thot_Raw, -ASR_Thot_T, -ASR_Attn_Raw, -ASR_Attn_T, -ASR_Aggr_Raw, -ASR_Aggr_T, -ASR_Rule_Raw, -ASR_Rule_T, -ASR_Intr_Raw, -ASR_Intr_T, -ASR_Oth_Raw, -ASR_Crit_Raw, -ASR_Intn_Raw, -ASR_Intn_T, -ASR_Extn_Raw, -ASR_Extn_T, -ASR_TAO_Sum, -ASR_Totp_Raw, -ASR_Totp_T, -DSM_Depr_Raw, -DSM_Depr_T, -DSM_Anxi_Raw, -DSM_Anxi_T, -DSM_Somp_Raw, -DSM_Somp_T, -DSM_Avoid_Raw, -DSM_Avoid_T, -DSM_Adh_Raw, -DSM_Adh_T, -DSM_Inat_Raw, -DSM_Hype_Raw, -DSM_Antis_Raw, -DSM_Antis_T, -SSAGA_ChildhoodConduct, -SSAGA_PanicDisorder, -SSAGA_Agoraphobia, -SSAGA_Depressive_Ep, -SSAGA_Depressive_Sx, -EVA_Denom, Correction, -Noise_Comp, Odor_Unadj, Odor_AgeAdj, -PainIntens_RawScore, -PainInterf_Tscore, -Taste_Unadj, -Taste_AgeAdj, Mars_Log_Score, -Mars_Errs, Mars_Final, -THC, -SSAGA_Times_Used_Illicits, -SSAGA_Times_Used_Cocaine, -SSAGA_Times_Used_Hallucinogens, -SSAGA_Times_Used_Opiates, -SSAGA_Times_Used_Sedatives, -SSAGA_Times_Used_Stimulants, -SSAGA_Mj_Use, -SSAGA_Mj_Ab_Dep, SSAGA_Mj_Age_1st_Use, -SSAGA_Mj_Times_Used, -Total_Drinks_7days, -Num_Days_Drank_7days, -Avg_Weekday_Drinks_7days, -Avg_Weekend_Drinks_7days, -Total_Beer_Wine_Cooler_7days, -Avg_Weekday_Beer_Wine_Cooler_7days, -Avg_Weekend_Beer_Wine_Cooler_7days, -Total_Wine_7days, -Avg_Weekday_Wine_7days, -Avg_Weekend_Wine_7days, -Total_Hard_Liquor_7days, -Avg_Weekday_Hard_Liquor_7days, -Avg_Weekend_Hard_Liquor_7days, -SSAGA_Alc_D4_Dp_Sx, -SSAGA_Alc_D4_Ab_Dx, -SSAGA_Alc_D4_Ab_Sx, -SSAGA_Alc_D4_Dp_Dx, -SSAGA_Alc_12_Drinks_Per_Day, SSAGA_Alc_12_Frq, SSAGA_Alc_12_Frq_5plus, SSAGA_Alc_12_Frq_Drk, -SSAGA_Alc_12_Max_Drinks, SSAGA_Alc_Age_1st_Use, -SSAGA_Alc_Hvy_Drinks_Per_Day, SSAGA_Alc_Hvy_Frq, SSAGA_Alc_Hvy_Frq_5plus, SSAGA_Alc_Hvy_Frq_Drk, -SSAGA_Alc_Hvy_Max_Drinks, -Total_Any_Tobacco_7days, -Times_Used_Any_Tobacco_Today, -Num_Days_Used_Any_Tobacco_7days, -Avg_Weekday_Any_Tobacco_7days, -Avg_Weekend_Any_Tobacco_7days, -Total_Cigarettes_7days, -Avg_Weekday_Cigarettes_7days, -Avg_Weekend_Cigarettes_7days, -SSAGA_TB_Smoking_History, -SSAGA_TB_Still_Smoking, MMSE_Score, -PSQI_Score, -PSQI_SleepQuality1, -PSQI_SleepLatency, -PSQI_SleepQuality2, -PSQI_SleepDuration, -PSQI_SleepDisturbance, -PSQI_SleepMeds, -PSQI_DayDysfunction, PicSeq_Unadj, PicSeq_AgeAdj, CardSort_Unadj, CardSort_AgeAdj, Flanker_Unadj, Flanker_AgeAdj, PMAT24_A_CR, -PMAT24_A_SI, -PMAT24_A_RTCR, ReadEng_Unadj, ReadEng_AgeAdj, PicVocab_Unadj, PicVocab_AgeAdj, ProcSpeed_Unadj, ProcSpeed_AgeAdj, DDisc_SV_1mo_200, DDisc_SV_6mo_200, DDisc_SV_1yr_200, DDisc_SV_3yr_200, DDisc_SV_5yr_200, DDisc_SV_10yr_200, DDisc_SV_1mo_40K, DDisc_SV_6mo_40K, DDisc_SV_1yr_40K, DDisc_SV_3yr_40K, DDisc_SV_5yr_40K, DDisc_SV_10yr_40K, DDisc_AUC_200, DDisc_AUC_40K, VSPLOT_TC, -VSPLOT_CRTE, -VSPLOT_OFF, SCPT_TP, SCPT_TN, -SCPT_FP, -SCPT_FN, -SCPT_TPRT, SCPT_SEN, SCPT_SPEC, -SCPT_LRNR, IWRD_TOT, -IWRD_RTC, ListSort_Unadj, ListSort_AgeAdj, ER40_CR, -ER40_CRT, ER40ANG, ER40FEAR, ER40NOE, ER40SAD, -AngAffect_Unadj, -AngHostil_Unadj, -AngAggr_Unadj, -FearAffect_Unadj, -FearSomat_Unadj, -Sadness_Unadj, LifeSatisf_Unadj, MeanPurp_Unadj, PosAffect_Unadj, Friendship_Unadj, -Loneliness_Unadj, -PercHostil_Unadj, -PercReject_Unadj, EmotSupp_Unadj, InstruSupp_Unadj, -PercStress_Unadj, SelfEff_Unadj, Endurance_Unadj, Endurance_AgeAdj, GaitSpeed_Comp, Dexterity_Unadj, Dexterity_AgeAdj, Strength_Unadj, Strength_AgeAdj, NEOFAC_A, NEOFAC_O, NEOFAC_C, NEOFAC_N, NEOFAC_E.

2 SIGN ALIGNMENT

2.1 Effects of Sign-flipping

LEMMA 1. *Let X be a column-mean-centred matrix of dimensions $N \times D$. Randomly flipping the signs of columns does not affect the subject-wise covariance matrix, and affects the variable-wise covariance matrix in a predictable way.*

PROOF. Flipping the signs of columns of X is equivalent with right-multiply a diagonal matrix R with -1 or 1 on diagonal. The subject-wise covariance is defined as $\frac{1}{D}XX^\top$. Therefore, flipping the column signs in subject-wise covariance gives

$$\frac{1}{D}(XR)(XR)^\top = \frac{1}{D}XRR^\top X^\top = \frac{1}{D}XX^\top. \tag{S1}$$

The variable-wise covariance is defined as $\frac{1}{N}X^\top X$. After flipping the column signs,

$$\frac{1}{N}(XR)^\top(XR) = \frac{1}{N}R^\top X^\top XR. \tag{S2}$$

This suggests the variable-wise covariance have corresponding rows and columns sign-flipped simultaneously, i.e. having predictable signs flipped at certain entries. ■

THEOREM 2. *Let X be a column-mean-centred matrix of dimensions $N \times D$. Randomly flipping the signs of columns does not change the principal components in PCA, and the principal loadings have the corresponding rows flipped.*

PROOF. We prove the above statements of PCA by showing the sign-flipping effects on Singular Value Decomposition (SVD). SVD has form

$$X = U\Sigma V^\top, \tag{S3}$$

where $U\Sigma$ is known as the principal components and V is the principal loadings. Therefore, from the proof of Lemma 1, flipping the column signs of X gives

$$XR = U\Sigma V^\top R = U\Sigma(RV)^\top. \tag{S4}$$

Therefore, it leaves the principal components un-changed and principal loadings have R left-multiplied to it which is equivalent with flipping the corresponding row signs. ■

THEOREM 3. *Suppose the inputs of CCA are X and Y. Both X and Y are column-mean-centred. Flipping column signs of X and/or Y does not change the canonical variables, but changes the signs of canonical loadings.*

PROOF. Let P and Q be canonical variables, A and B be canonical weights for X and Y respectively (as described in Section 2.4). We have

$$\begin{aligned} P &= XA, \\ Q &= YB. \end{aligned} \tag{S5}$$

We prove the case for flipping columns of X only, and the case for Y can be proved similarly. The solution for A are the eigenvectors of $U = C^{-1}_{(X,X)}C_{(X,Y)}C^{-1}_{(Y,Y)}C_{(Y,X)}$, where C stands for the covariance matrix. Now we show that flipping column signs of X would affect A by flipping the respective row signs. We ignore all scaling factors in the covariance matrices. Assume R be the sign-flipping diagonal matrix.

$$\begin{aligned} C^{-1}_{(XR,XR)}C_{(XR,Y)}C^{-1}_{(Y,Y)}C_{(Y,XR)} &= (RX^\top XR)^{-1} \cdot (RX^\top Y) \cdot (Y^\top Y)^{-1} \cdot (Y^\top XR) \\ &= R(X^\top X)^{-1}R \cdot (RX^\top Y) \cdot (Y^\top Y)^{-1} \cdot (Y^\top XR) \\ &= R \cdot C^{-1}_{(X,X)}C_{(X,Y)}C^{-1}_{(Y,Y)}C_{(Y,X)} \cdot R \\ &= RUR. \end{aligned} \tag{S6}$$

The eigen-decomposition of RUR is (ignoring the scaling factor)

$$(RUR)^\top RUR = RU^\top UR = RA\Lambda(RA)^\top \tag{S7}$$

Therefore, by flipping the column signs of X, the canonical weights A change to RA, and the canonical variables P remain unchanged ($P = XRRA$).

The canonical loadings for X are the Pearson's correlations between X and columns of P. Thus, by flipping the columns of X, we have the canonical loadings sign-flipped for the corresponding variables. ■

2.2 Correlation Matrix

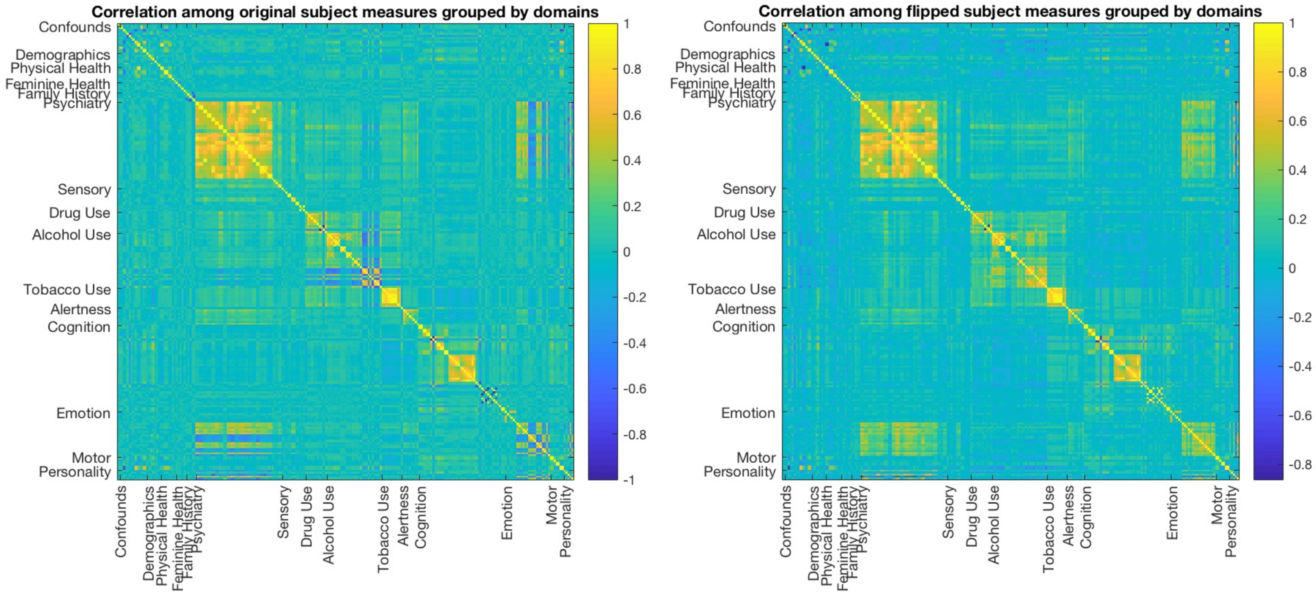

Figure S.1. Pairwise correlation among 234 subject measures grouped by 14 functional domains. On the left is the correlation matrix among original variables; on the right is the correlation matrix after sign-flipping which aims to align pairwise correlations between and within domains. Alcohol Use and Emotion sub-domains have the most noticeable changes, reflected by the within domain correlation pattern on the diagonal. However, almost all variables in Tobacco Use and Psychiatry are sign-flipped as well. Flipping all variables within a sub-domain preserves the within-domain correlation pattern. The change is reflected by the correlation with variables in other sub-domains.

3 CONFOUNDERS

Confounders include: data release, data acquisition, gender, age (and age^2), race white (binary), race black (binary), other race (binary), ethnicity, height (and height^2), weight (and weight^2), BMI, 3T fMRI Reconstruction Version, head motion, intra-cranial volume (cubed) and brain segmentation volume (cubed).

4 CANONICAL LOADINGS FOR PCA CCA

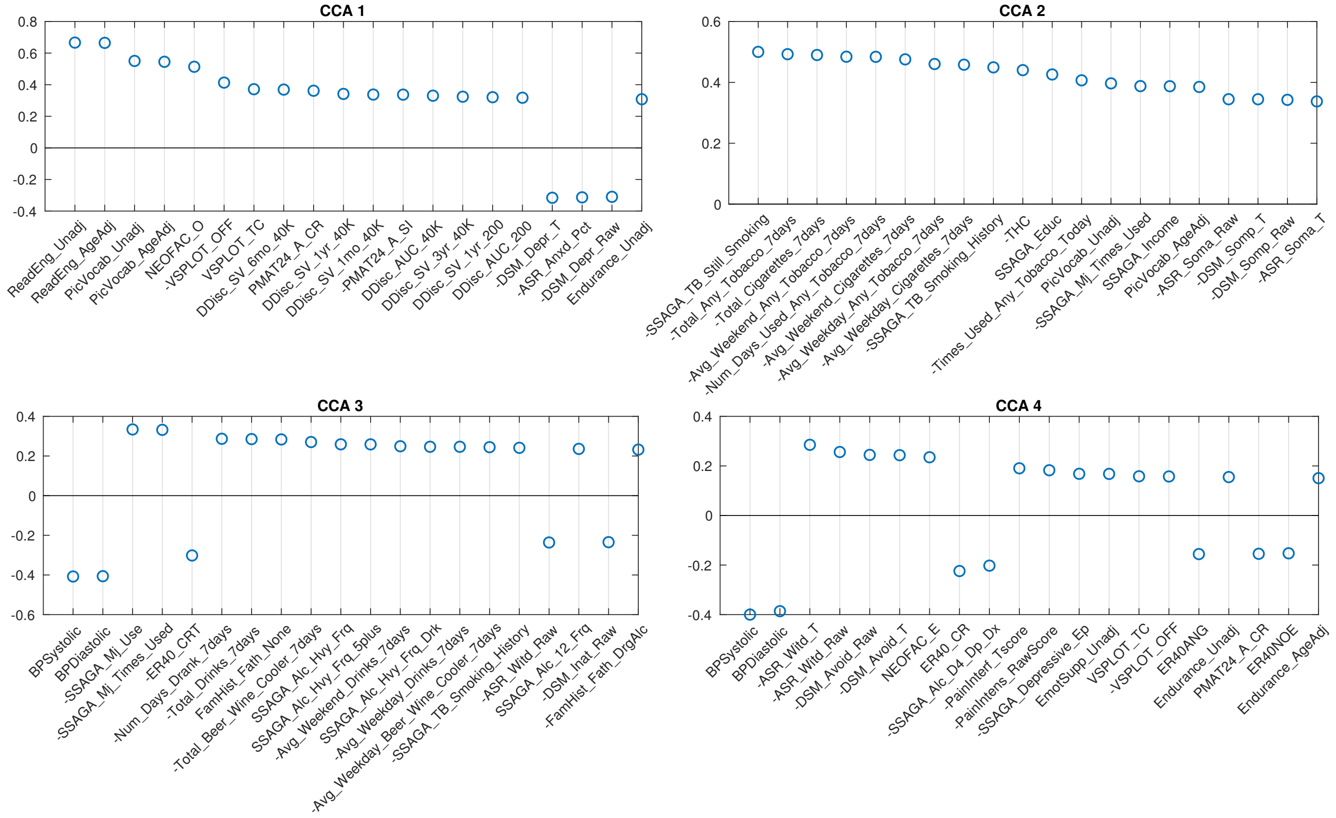

Figure S.2. Top 20 canonical loadings for 4 significant SM canonical variables in PCA CCA method using 62 dimensional SM and 100 dimensional BM. Variable names with a '-' sign means the values have been flipped.

5 SUMMARY REPORTS

In the following sub-domain reports, by cross-validating PCs within each sub-domain, we observe strong stability on the PCs: PCs in test set explain almost the same amount of variance with the ones in the training set (panel B). This also implies the stability of DDR. We further investigated the principal loadings for the number of DDR estimated dimensions, and found with factor rotation, principal loadings become more interpretable (panel D and E). Therefore, we summarized the meaning of each rotated factor by panel E in the summary reports, and their names are listed in the middle column of Tab. 2.

Moreover, the null eigen-spectrum (green dots in panel A) as introduced in Section 2.3, shows that for example, in the 'Family History' sub-domain, only two latent dimensions explain more variance than the 'background noise'. This is consistent with the DDR estimation for the dimension of 'Family History' (panel F, Fig. S.3), and this is the roughly the case for all sub-domains.

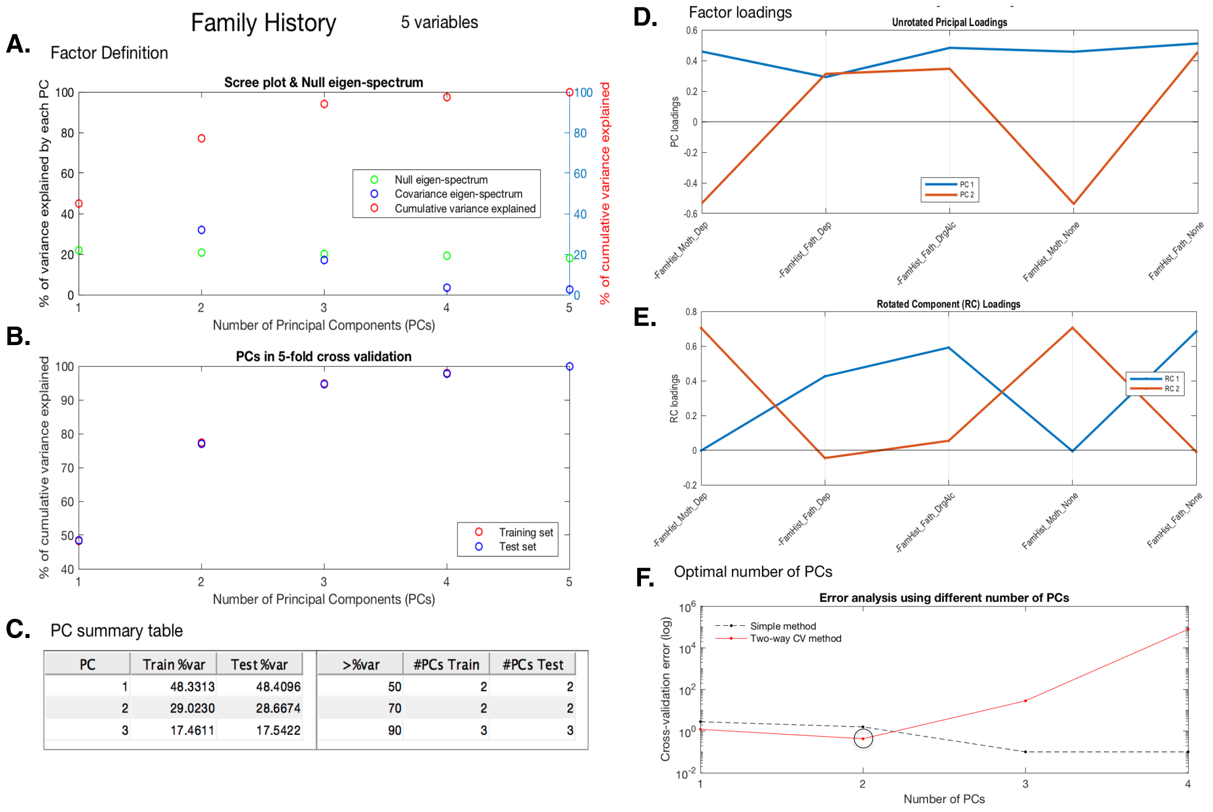

PC	Train %var	Test %var	>%var	#PCs Train	#PCs Test
1	48.3313	48.4096	50	2	2
2	29.0230	28.6674	70	2	2
3	17.4611	17.5422	90	3	3

Figure S.3. Summary report of Family History. Panel A shows the eigen-spectrum (blue), cumulative eigen-spectrum (red) and null eigen-spectrum (green); panel B shows the cumulative variance explained by principal components (PCs) in cross-validation; panel C is the summary table for panel B showing 3 benchmark percentages 50%, 70% and 90%; panel D shows the principal loadings for optimal number of PCs; panel E shows the rotated loadings in D; panel F shows the error curves calculated by Eqn.6 and Eqn.8, with the minimal error circled at the second component. The naive way of calculating PRESS (dotted line) is monotonically decreasing, while the two-way CV method (red line) offers a minimum point at the second PC (circled).

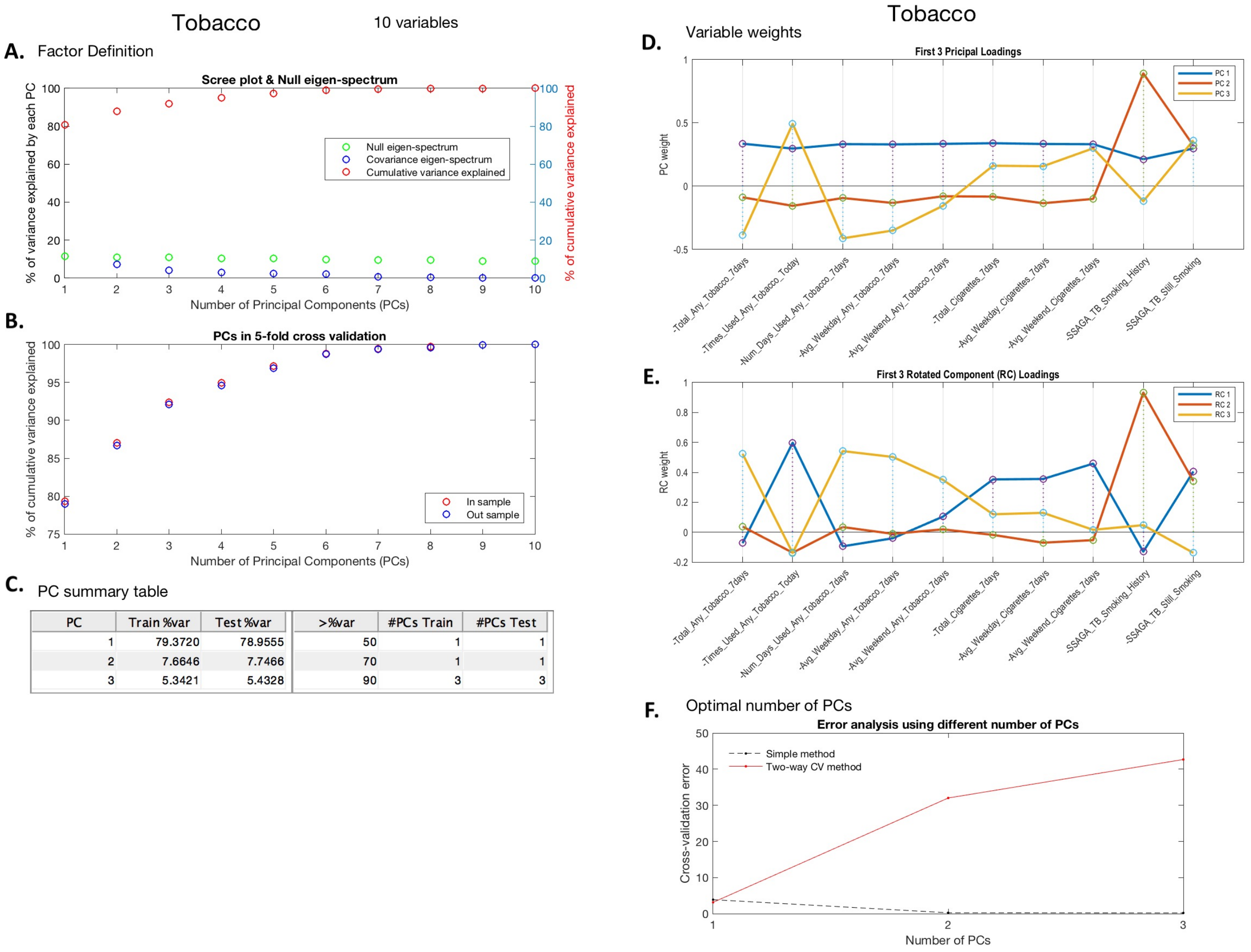

PC	Train %var	Test %var	>%var	#PCs Train	#PCs Test
1	79.3720	78.9555	50	1	1
2	7.6646	7.7466	70	1	1
3	5.3421	5.4328	90	3	3

Figure S.4. Tobacco Use sub-domain summary report. Panel A shows the eigen-spectrum (blue), cumulative eigen-spectrum (red) and null eigen-spectrum (green); panel B shows the cumulative variance explained by principal components (PCs) in cross-validation; panel C is the summary table for panel B showing 3 benchmark percentages 50%, 70% and 90%; panel D shows the principal loadings for optimal number of PCs; panel E shows the rotated loadings in D; panel F shows the error curves calculated by Eqn.6 and Eqn.8, with the minimal error circled at the second component. The naive way of calculating PRESS (dotted line) is monotonically decreasing, while the two-way CV method (red line) offers a minimum point.

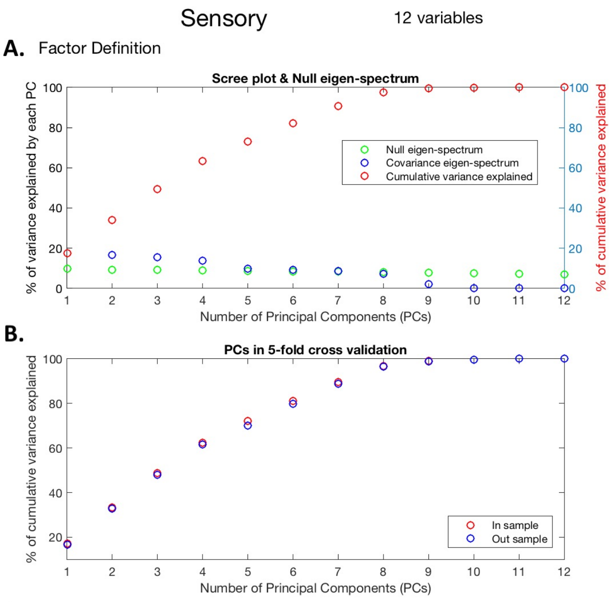

C. PC summary table

PC	Train %var	Test %var	>%var	#PCs Train	#PCs Test
1	17.2421	16.6426	50	4	4
2	16.1710	16.1049	70	4	5
3	15.4144	15.3310	90	8	8

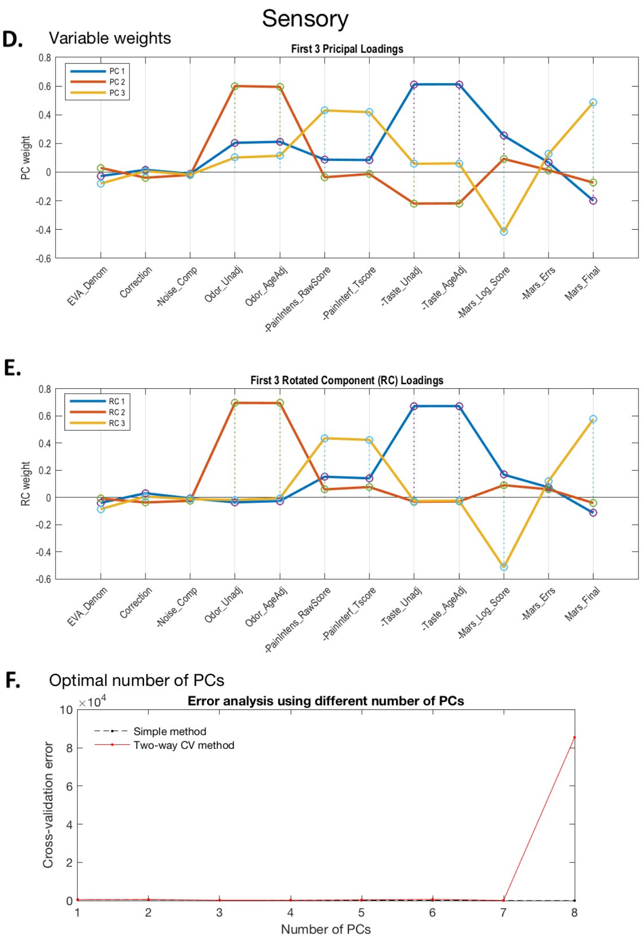

Figure S.5. Sensory sub-domain summary report. Panel A shows the eigen-spectrum (blue), cumulative eigen-spectrum (red) and null eigen-spectrum (green); panel B shows the cumulative variance explained by principal components (PCs) in cross-validation; panel C is the summary table for panel B showing 3 benchmark percentages 50%, 70% and 90%; panel D shows the principal loadings for optimal number of PCs; panel E shows the rotated loadings in D; panel F shows the error curves calculated by Eqn.6 and Eqn.8, with the minimal error circled at the second component. The naive way of calculating PRESS (dotted line) is monotonically decreasing, while the two-way CV method (red line) offers a minimum point.

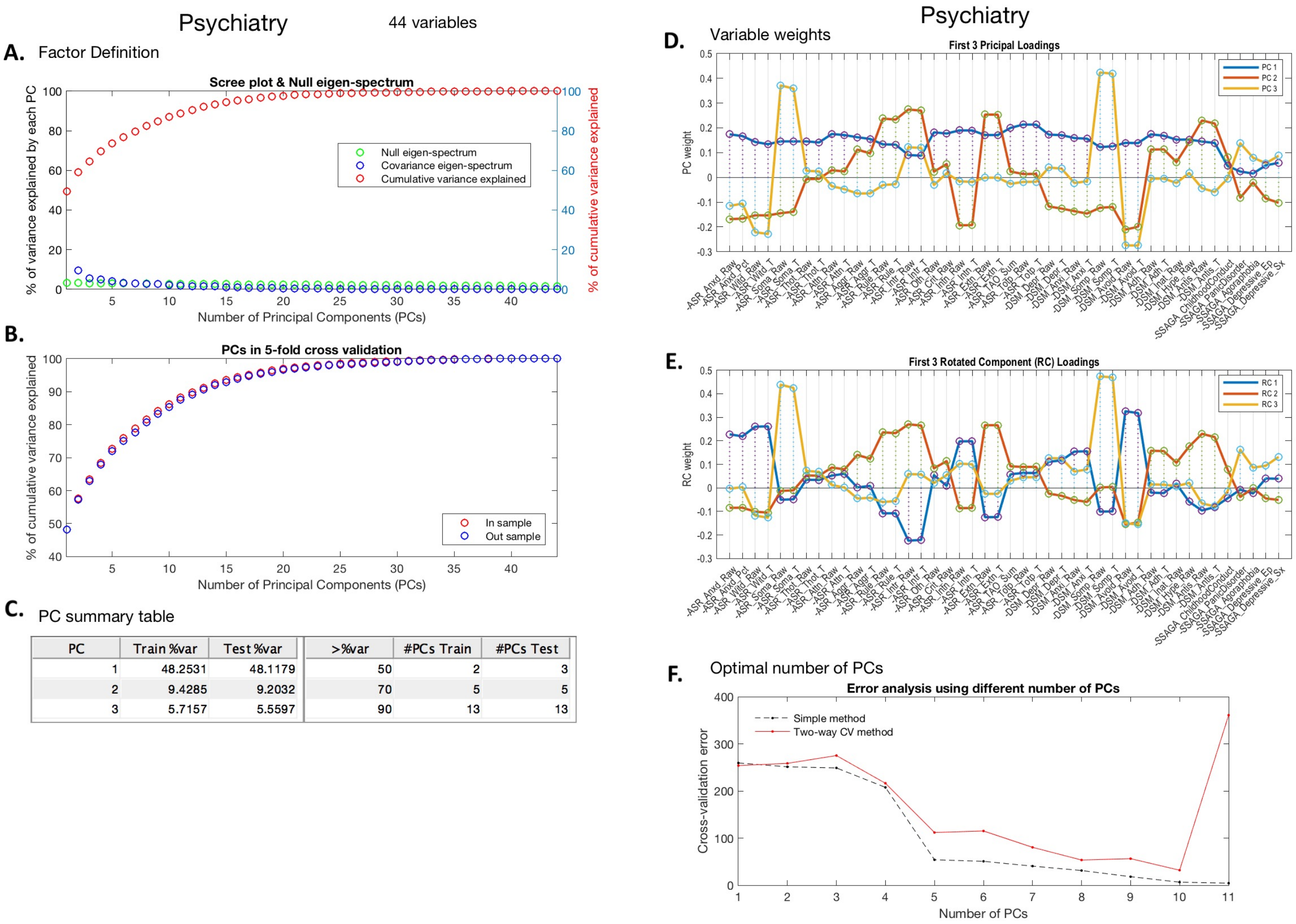

PC	Train %var	Test %var	>%var	#PCs Train	#PCs Test
1	48.2531	48.1179	50	2	3
2	9.4285	9.2032	70	5	5
3	5.7157	5.5597	90	13	13

Figure S.6. Psychiatry sub-domain summary report. Panel A shows the eigen-spectrum (blue), cumulative eigen-spectrum (red) and null eigen-spectrum (green); panel B shows the cumulative variance explained by principal components (PCs) in cross-validation; panel C is the summary table for panel B showing 3 benchmark percentages 50%, 70% and 90%; panel D shows the principal loadings for optimal number of PCs; panel E shows the rotated loadings in D; panel F shows the error curves calculated by Eqn.6 and Eqn.8, with the minimal error circled at the second component. The naive way of calculating PRESS (dotted line) is monotonically decreasing, while the two-way CV method (red line) offers a minimum point.

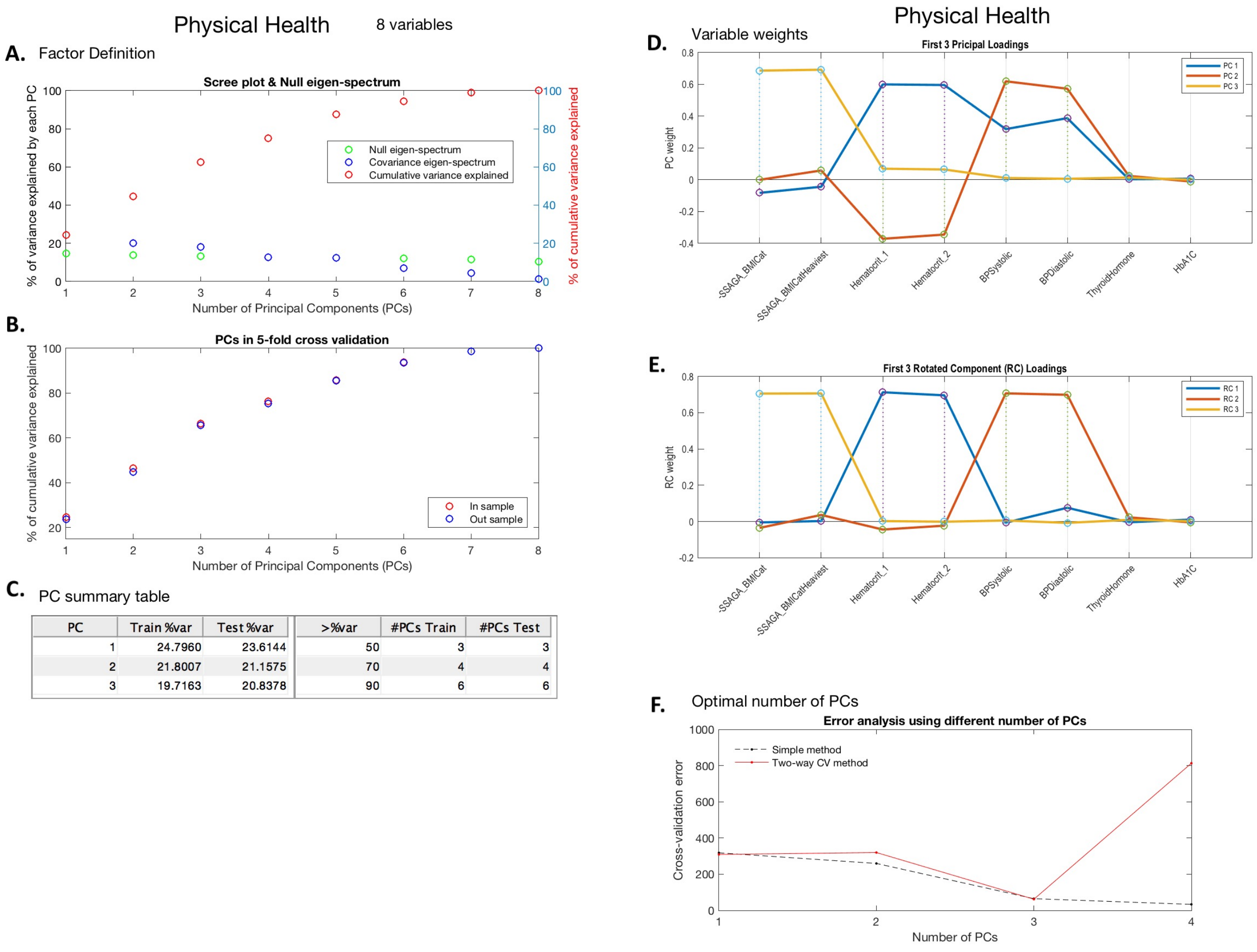

PC	Train %var	Test %var	>%var	#PCs Train	#PCs Test
1	24.7960	23.6144	50	3	3
2	21.8007	21.1575	70	4	4
3	19.7163	20.8378	90	6	6

Figure S.7. Physical Health sub-domain summary report. Panel A shows the eigen-spectrum (blue), cumulative eigen-spectrum (red) and null eigen-spectrum (green); panel B shows the cumulative variance explained by principal components (PCs) in cross-validation; panel C is the summary table for panel B showing 3 benchmark percentages 50%, 70% and 90%; panel D shows the principal loadings for optimal number of PCs; panel E shows the rotated loadings in D; panel F shows the error curves calculated by Eqn.6 and Eqn.8, with the minimal error circled at the second component. The naive way of calculating PRESS (dotted line) is monotonically decreasing, while the two-way CV method (red line) offers a minimum point.

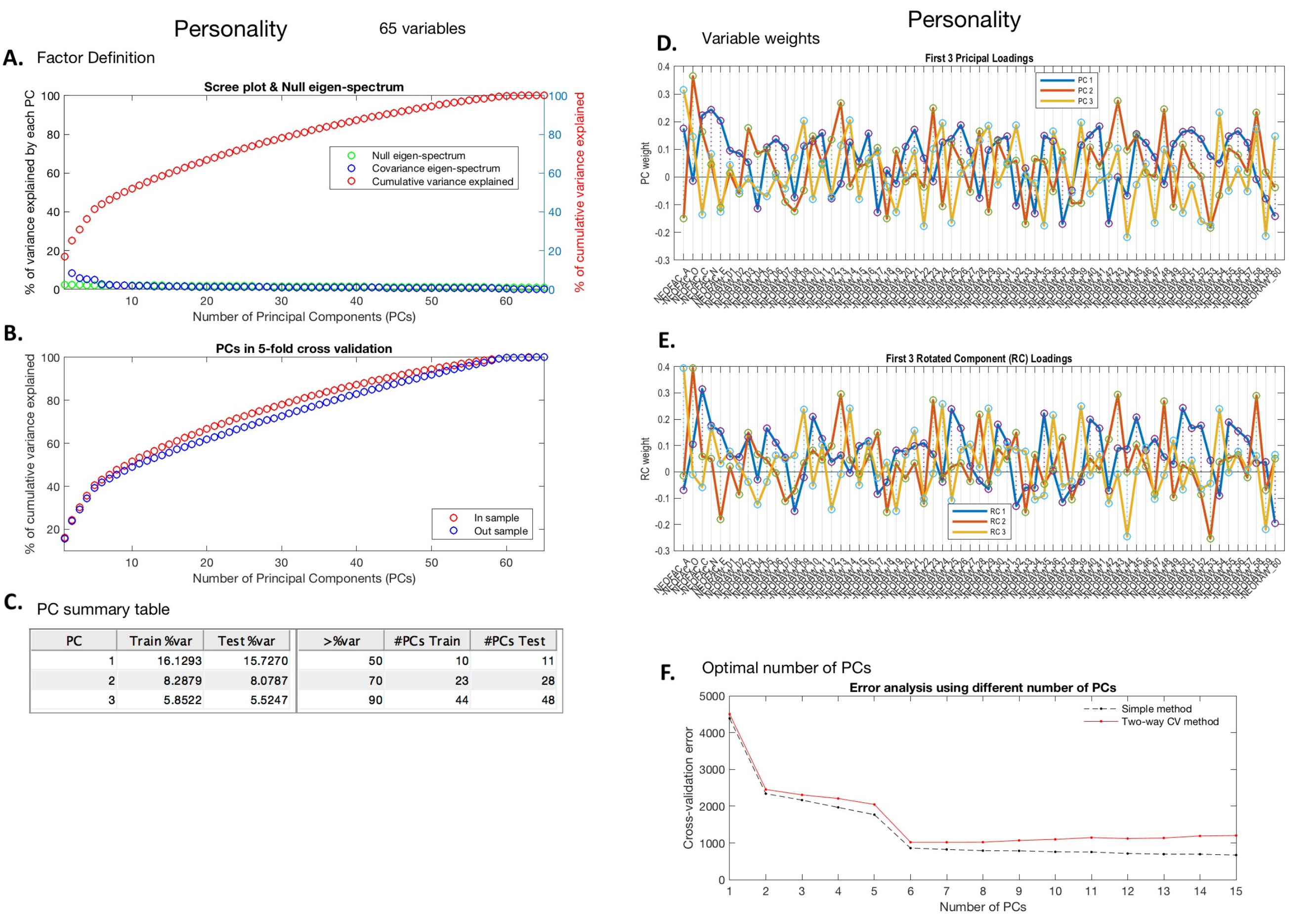

PC	Train %var	Test %var	>%var	#PCs Train	#PCs Test
1	16.1293	15.7270	50	10	11
2	8.2879	8.0787	70	23	28
3	5.8522	5.5247	90	44	48

Figure S.8. Personality sub-domain summary report. Panel A shows the eigen-spectrum (blue), cumulative eigen-spectrum (red) and null eigen-spectrum (green); panel B shows the cumulative variance explained by principal components (PCs) in cross-validation; panel C is the summary table for panel B showing 3 benchmark percentages 50%, 70% and 90%; panel D shows the principal loadings for optimal number of PCs; panel E shows the rotated loadings in D; panel F shows the error curves calculated by Eqn.6 and Eqn.8, with the minimal error circled at the second component. The naive way of calculating PRESS (dotted line) is monotonically decreasing, while the two-way CV method (red line) offers a minimum point.

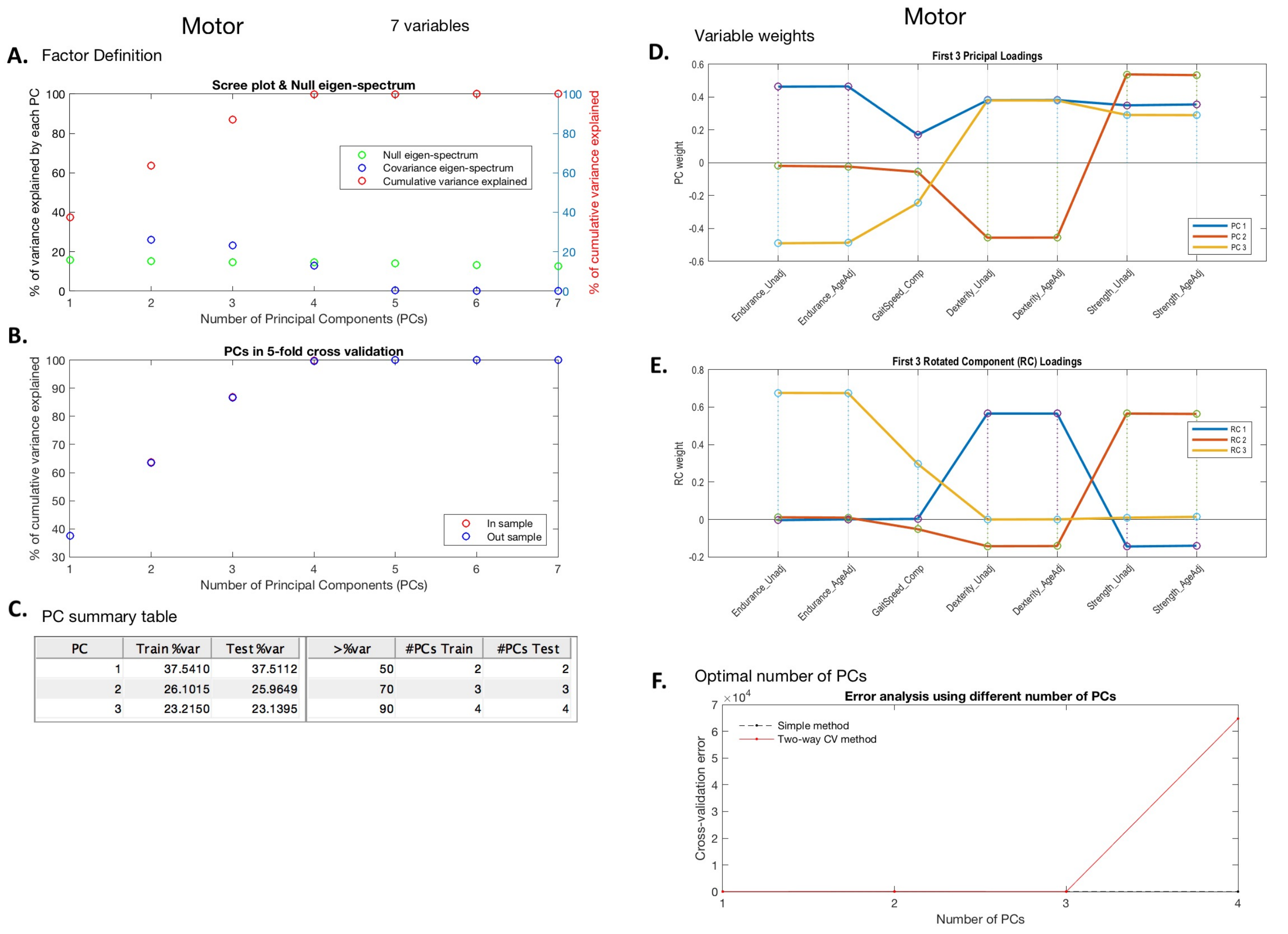

Figure S.9. Motor sub-domain summary report. Panel A shows the eigen-spectrum (blue), cumulative eigen-spectrum (red) and null eigen-spectrum (green); panel B shows the cumulative variance explained by principal components (PCs) in cross-validation; panel C is the summary table for panel B showing 3 benchmark percentages 50%, 70% and 90%; panel D shows the principal loadings for optimal number of PCs; panel E shows the rotated loadings in D; panel F shows the error curves calculated by Eqn.6 and Eqn.8, with the minimal error circled at the second component. The naive way of calculating PRESS (dotted line) is monotonically decreasing, while the two-way CV method (red line) offers a minimum point.

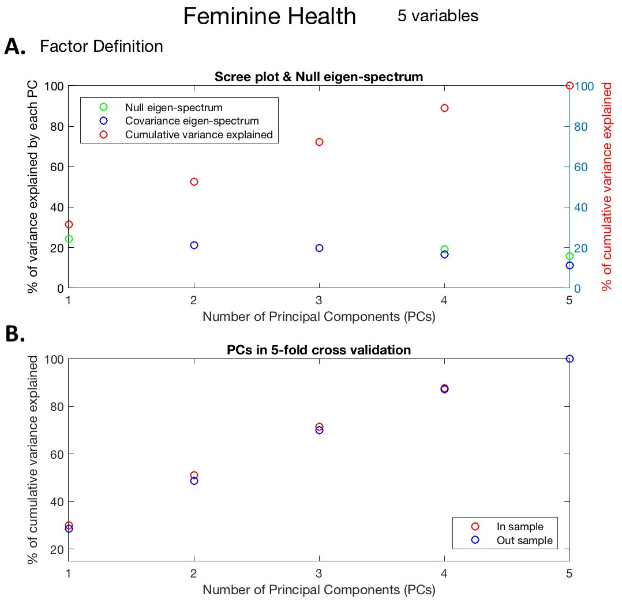

C. PC summary table

PC	Train %var	Test %var	>%var	#PCs Train	#PCs Test
1	30.0619	28.5554	50	2	3
2	21.0743	20.1500	70	3	3
3	20.2286	21.3870	90	5	5

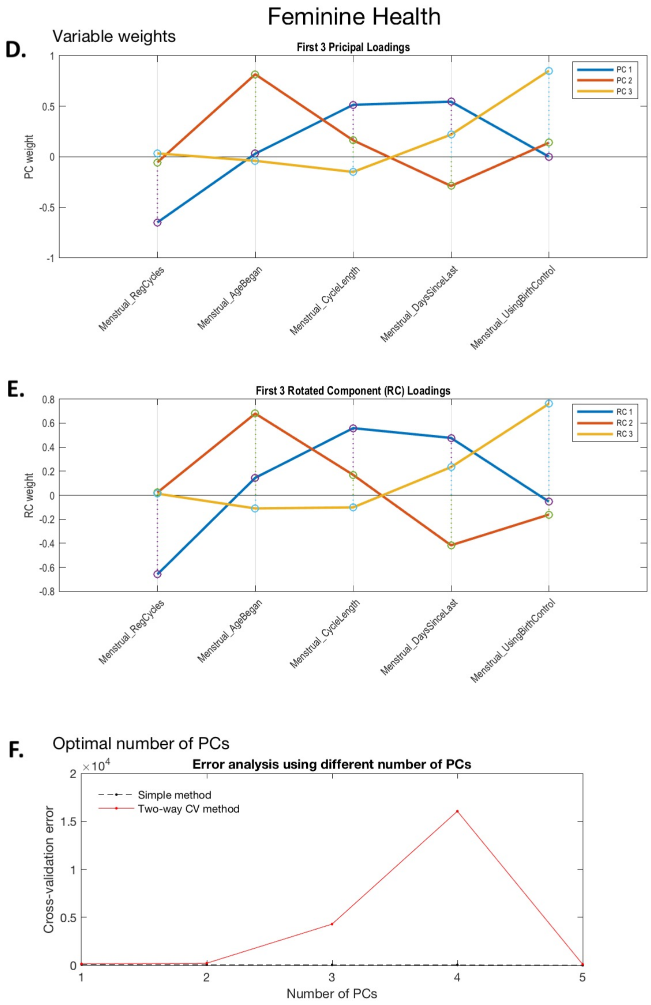

Figure S.10. Female Health sub-domain summary report. Notably this sub-domain is generated from female subjects only. Panel A shows the eigen-spectrum (blue), cumulative eigen-spectrum (red) and null eigen-spectrum (green); panel B shows the cumulative variance explained by principal components (PCs) in cross-validation; panel C is the summary table for panel B showing 3 benchmark percentages 50%, 70% and 90%; panel D shows the principal loadings for optimal number of PCs; panel E shows the rotated loadings in D; panel F shows the error curves calculated by Eqn.6 and Eqn.8, with the minimal error circled at the second component. The naive way of calculating PRESS (dotted line) is monotonically decreasing, while the two-way CV method (red line) offers a minimum point.

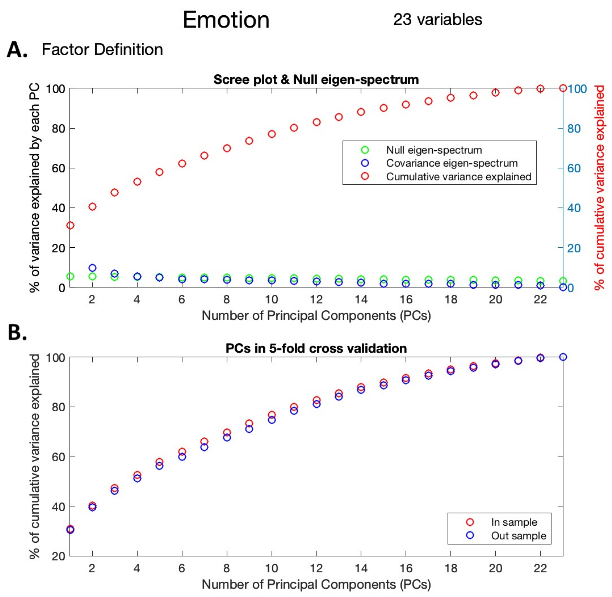

C. PC summary table

PC	Train %var	Test %var	>%var	#PCs Train	#PCs Test
1	30.9127	30.4036	50	4	4
2	9.4701	9.2103	70	9	9
3	6.8737	6.5393	90	16	16

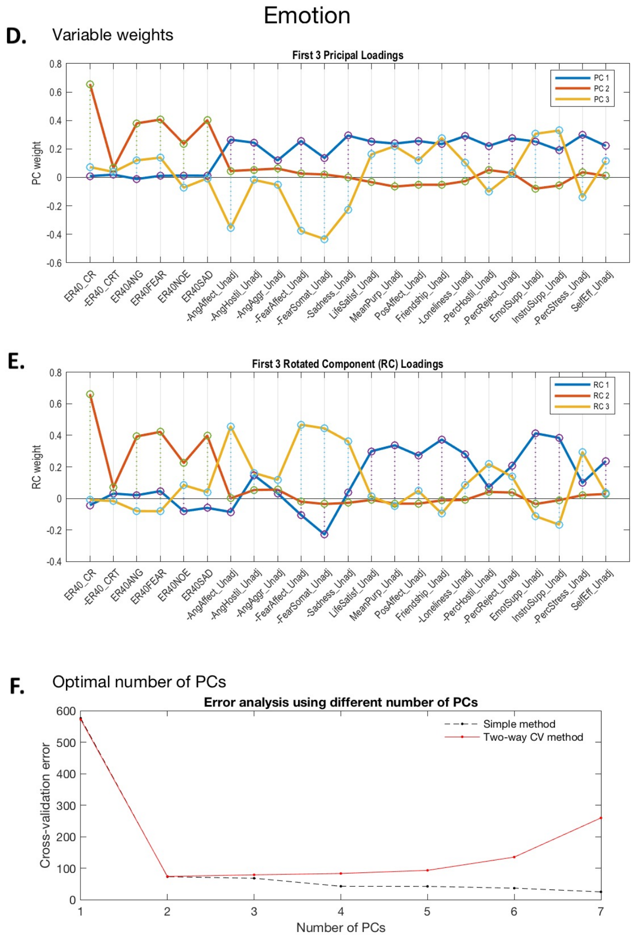

Figure S.11. Emotion sub-domain summary report. Panel A shows the eigen-spectrum (blue), cumulative eigen-spectrum (red) and null eigen-spectrum (green); panel B shows the cumulative variance explained by principal components (PCs) in cross-validation; panel C is the summary table for panel B showing 3 benchmark percentages 50%, 70% and 90%; panel D shows the principal loadings for optimal number of PCs; panel E shows the rotated loadings in D; panel F shows the error curves calculated by Eqn.6 and Eqn.8, with the minimal error circled at the second component. The naive way of calculating PRESS (dotted line) is monotonically decreasing, while the two-way CV method (red line) offers a minimum point.

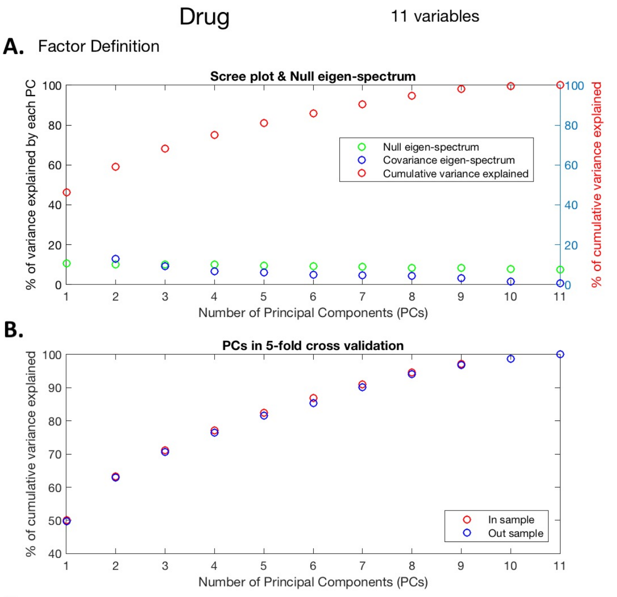

C. PC summary table

PC	Train %var	Test %var	>%var	#PCs Train	#PCs Test
1	50.0323	49.6622	50	1	2
2	13.1838	13.2278	70	3	3
3	7.8691	7.7633	90	7	7

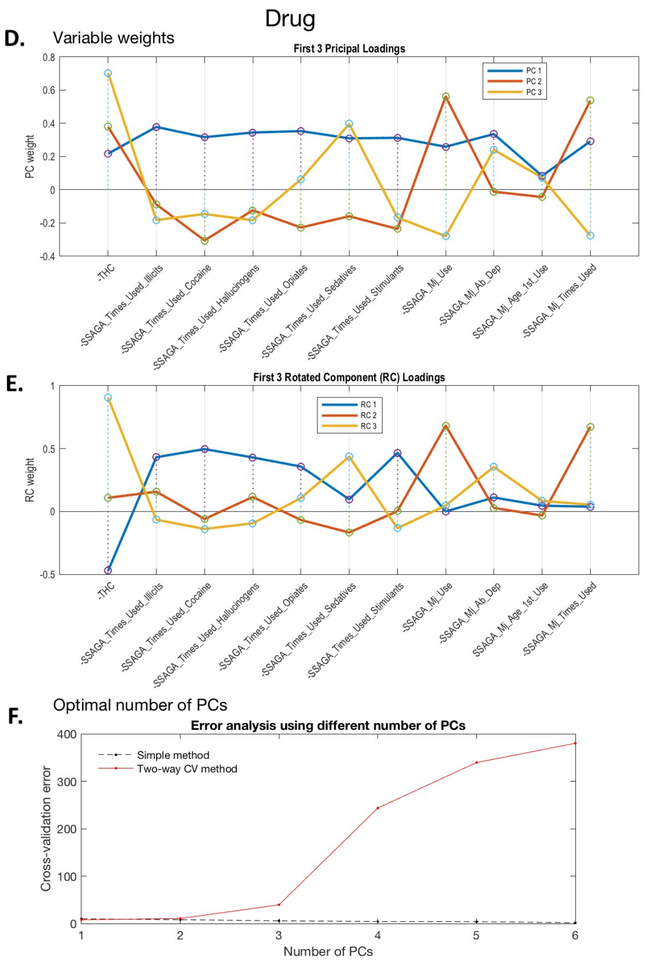

Figure S.12. Drug Use sub-domain summary report. Panel A shows the eigen-spectrum (blue), cumulative eigen-spectrum (red) and null eigen-spectrum (green); panel B shows the cumulative variance explained by principal components (PCs) in cross-validation; panel C is the summary table for panel B showing 3 benchmark percentages 50%, 70% and 90%; panel D shows the principal loadings for optimal number of PCs; panel E shows the rotated loadings in D; panel F shows the error curves calculated by Eqn.6 and Eqn.8, with the minimal error circled at the second component. The naive way of calculating PRESS (dotted line) is monotonically decreasing, while the two-way CV method (red line) offers a minimum point.

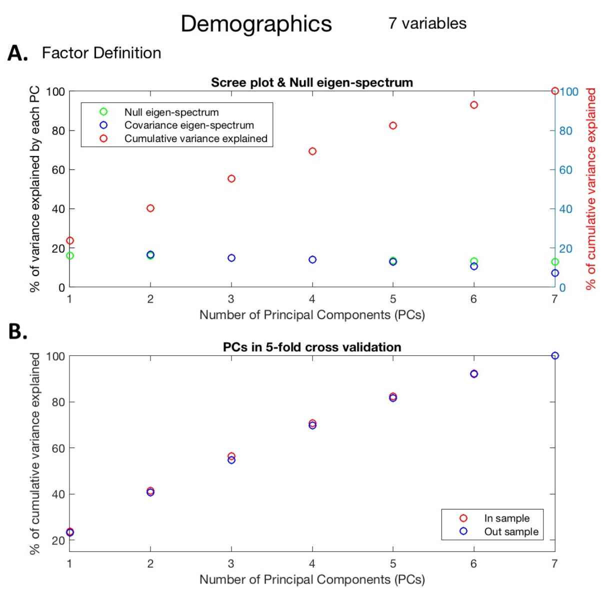

C. PC summary table

PC	Train %var	Test %var	>%var	#PCs Train	#PCs Test
1	23.8066	23.3390	50	3	3
2	17.6491	17.2493	70	4	5
3	14.9746	14.1821	90	6	6

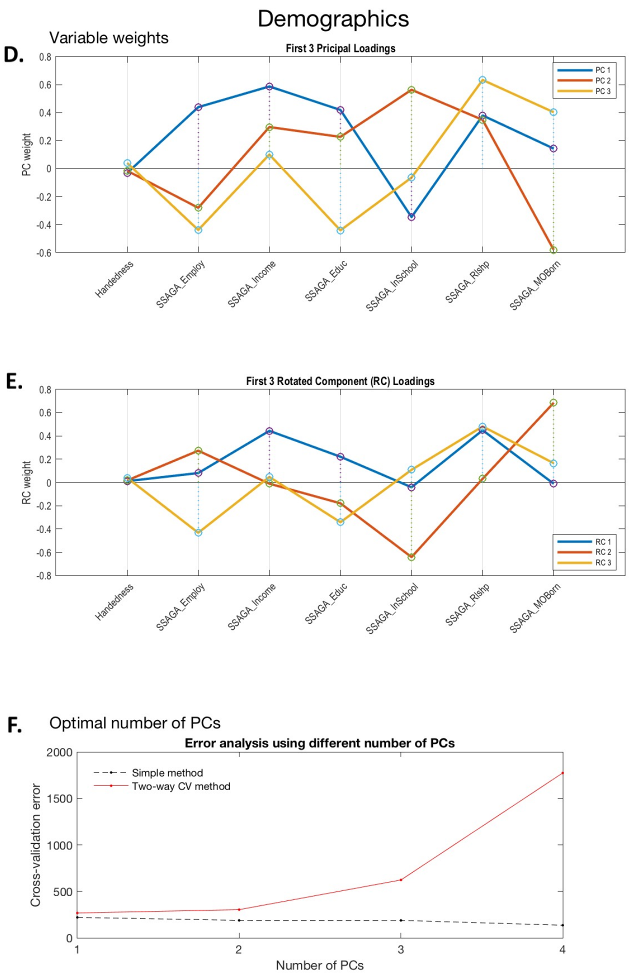

Figure S.13. Demographics and SES sub-domain summary report. Panel A shows the eigen-spectrum (blue), cumulative eigen-spectrum (red) and null eigen-spectrum (green); panel B shows the cumulative variance explained by principal components (PCs) in cross-validation; panel C is the summary table for panel B showing 3 benchmark percentages 50%, 70% and 90%; panel D shows the principal loadings for optimal number of PCs; panel E shows the rotated loadings in D; panel F shows the error curves calculated by Eqn.6 and Eqn.8, with the minimal error circled at the second component. The naive way of calculating PRESS (dotted line) is monotonically decreasing, while the two-way CV method (red line) offers a minimum point.

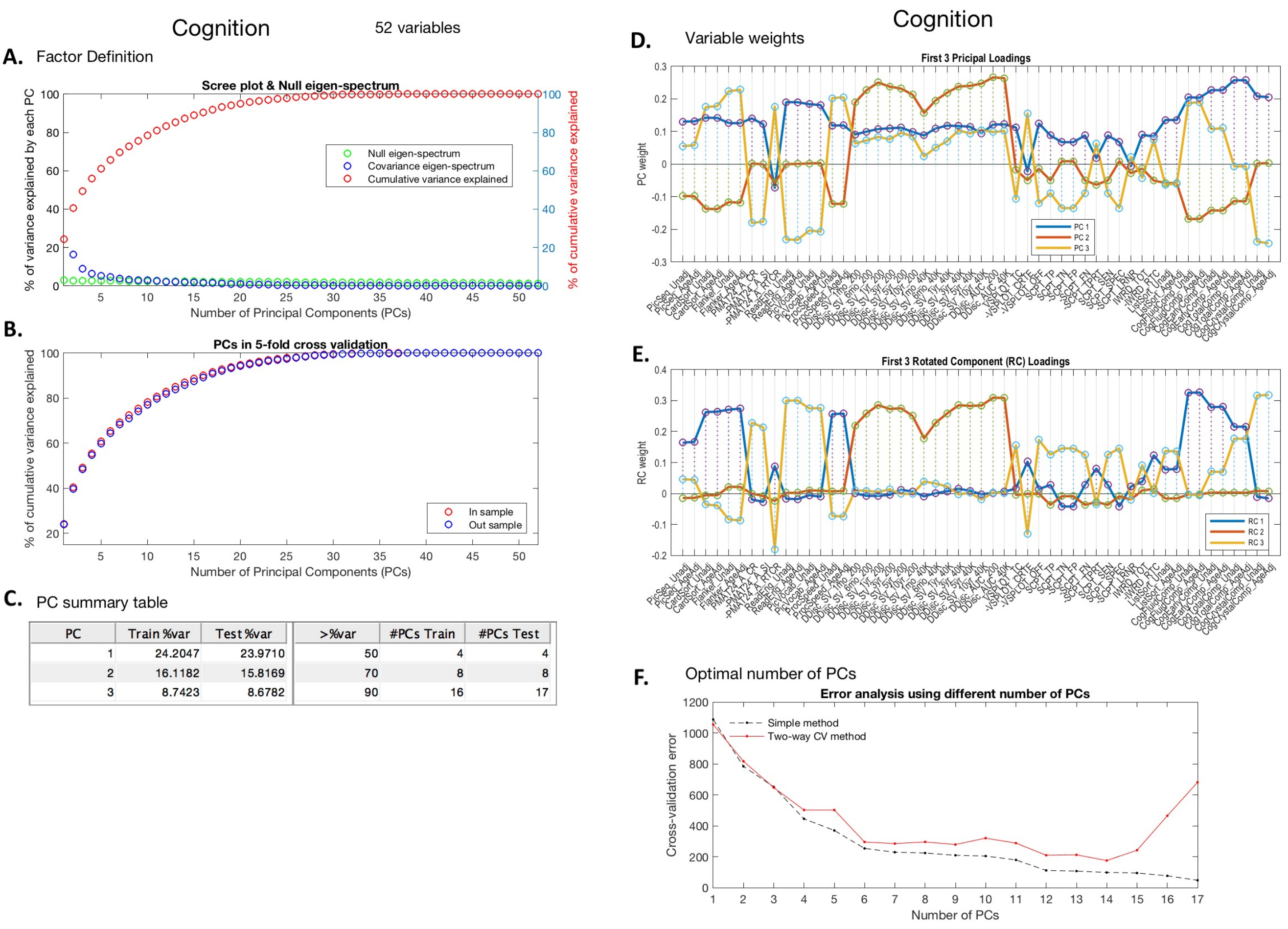

PC	Train %var	Test %var	>%var	#PCs Train	#PCs Test
1	24.2047	23.9710	50	4	4
2	16.1182	15.8169	70	8	8
3	8.7423	8.6782	90	16	17

Figure S.14. Cognition sub-domain summary report. Panel A shows the eigen-spectrum (blue), cumulative eigen-spectrum (red) and null eigen-spectrum (green); panel B shows the cumulative variance explained by principal components (PCs) in cross-validation; panel C is the summary table for panel B showing 3 benchmark percentages 50%, 70% and 90%; panel D shows the principal loadings for optimal number of PCs; panel E shows the rotated loadings in D; panel F shows the error curves calculated by Eqn.6 and Eqn.8, with the minimal error circled at the second component. The naive way of calculating PRESS (dotted line) is monotonically decreasing, while the two-way CV method (red line) offers a minimum point.

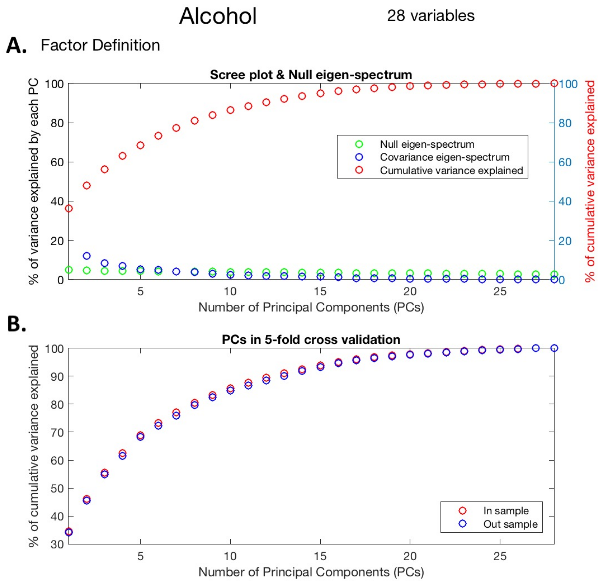

C. PC summary table

PC	Train %var	Test %var	>%var	#PCs Train	#PCs Test
1	34.5595	34.2072	50	3	3
2	11.5472	11.3400	70	6	6
3	9.5121	9.4337	90	13	14

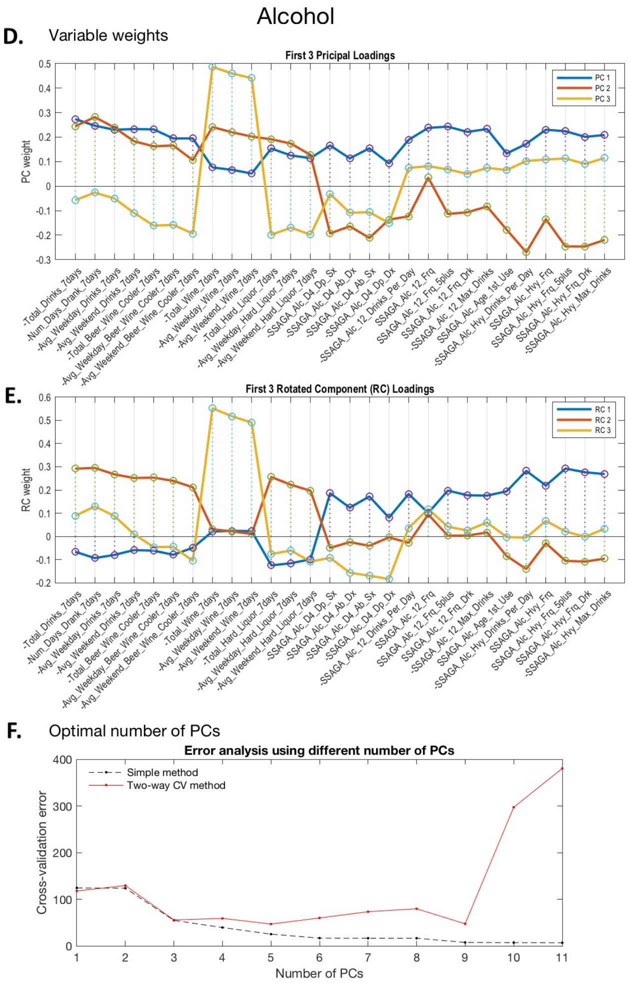

Figure S.15. Alcohol Use sub-domain summary report. Panel A shows the eigen-spectrum (blue), cumulative eigen-spectrum (red) and null eigen-spectrum (green); panel B shows the cumulative variance explained by principal components (PCs) in cross-validation; panel C is the summary table for panel B showing 3 benchmark percentages 50%, 70% and 90%; panel D shows the principal loadings for optimal number of PCs; panel E shows the rotated loadings in D; panel F shows the error curves calculated by Eqn.6 and Eqn.8, with the minimal error circled at the second component. The naive way of calculating PRESS (dotted line) is monotonically decreasing, while the two-way CV method (red line) offers a minimum point.

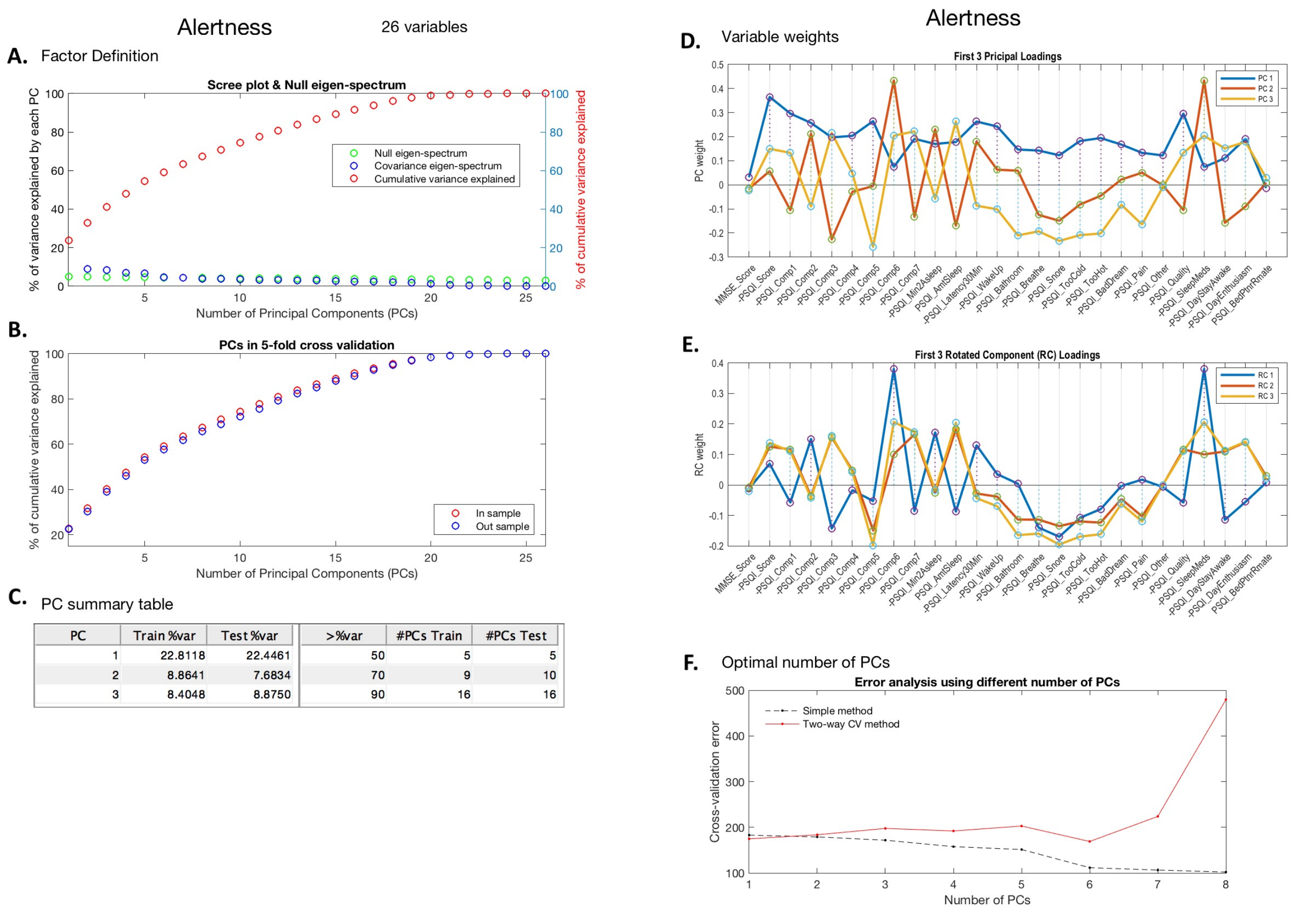

PC	Train %var	Test %var	>%var	#PCs Train	#PCs Test
1	22.8118	22.4461	50	5	5
2	8.8641	7.6834	70	9	10
3	8.4048	8.8750	90	16	16

Figure S.16. Alertness sub-domain summary report. Panel A shows the eigen-spectrum (blue), cumulative eigen-spectrum (red) and null eigen-spectrum (green); panel B shows the cumulative variance explained by principal components (PCs) in cross-validation; panel C is the summary table for panel B showing 3 benchmark percentages 50%, 70% and 90%; panel D shows the principal loadings for optimal number of PCs; panel E shows the rotated loadings in D; panel F shows the error curves calculated by Eqn.6 and Eqn.8, with the minimal error circled at the second component. The naive way of calculating PRESS (dotted line) is monotonically decreasing, while the two-way CV method (red line) offers a minimum point.

6 STABILITY OF CANONICAL LOADINGS

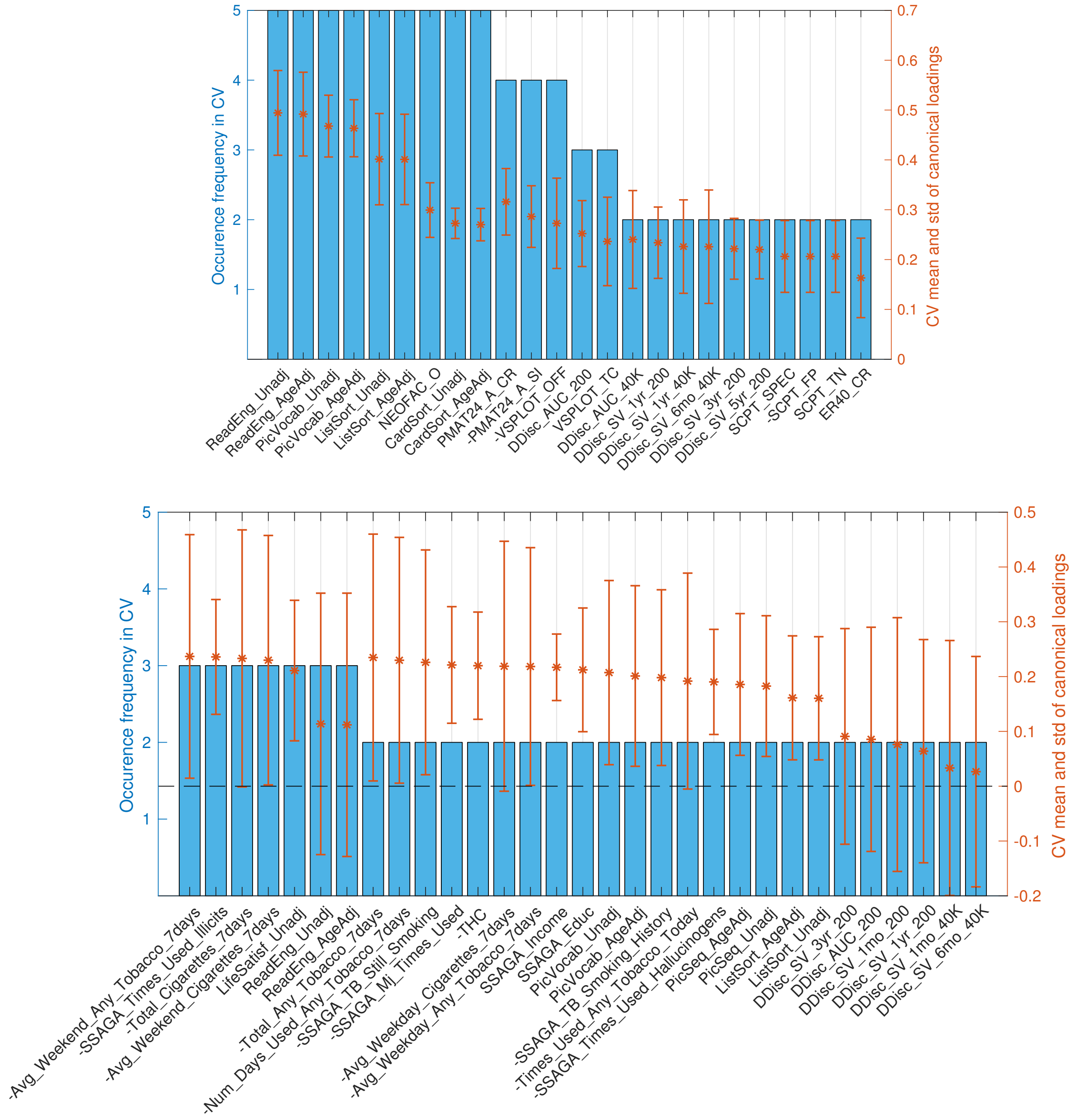

Figure S.17. Stability of SM canonical loadings on observed variables. Bar plot shows the occurrence frequency in CV out of the 5 folds. Variables are chosen by selecting the top 20 mostly weighted ones in each fold. The ones appeared at least twice are shown above. Right axis shows the mean and the standard deviation over all occurred loadings. Top and bottom plots are the canonical loadings for the first and second canonical variables respectively. It is obvious that the second canonical loadings are less stable than the first set.

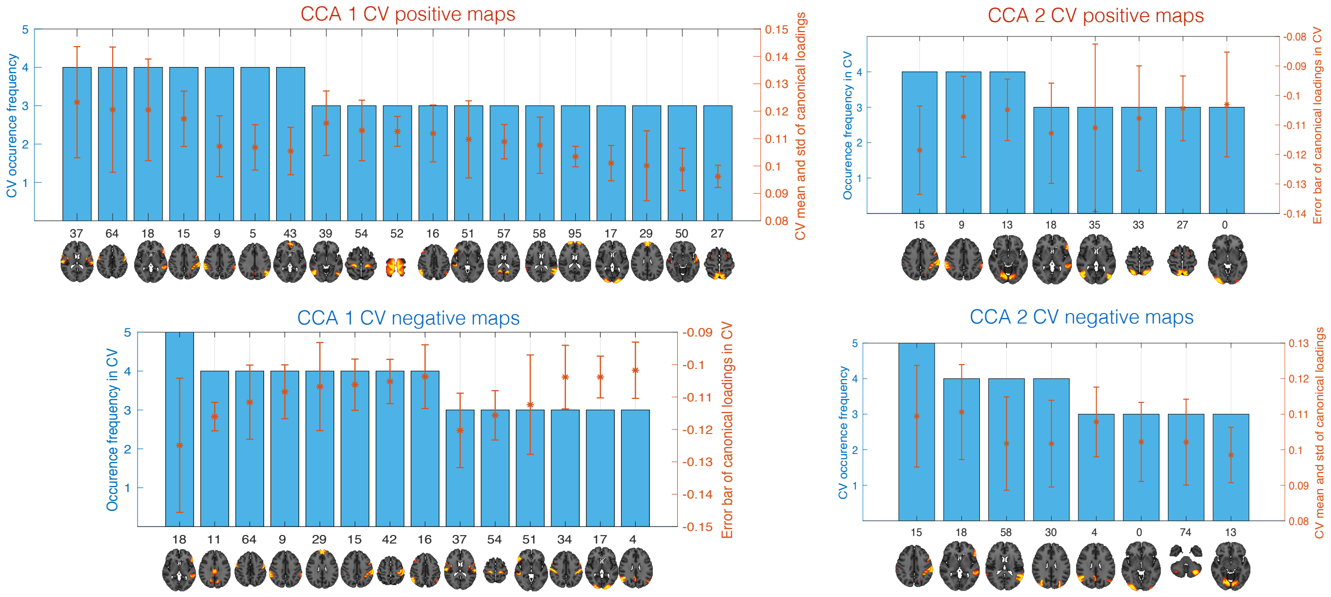

Figure S.18. Stability of BM canonical loadings on observed data. Bar plot shows the occurrence frequency in CV out of the 5 folds. The positive (top plots) and negative (bottom plots) maps are chosen by first averaging the top 20 positive and negative canonical loadings within each region respectively; then select the top 20 nodes with the highest positive and negative mean loadings in each fold. The ones occurred at least three times are shown above. Right axis shows the mean and the standard deviation over all occurred loadings. Similar to SM canonical loadings, the first set shows better stability than the second set.
